# A deep learning-based model for automatic identification of mesopelagic organisms from in-trawl cameras

**DOI:** 10.1371/journal.pone.0340640

**Published:** 2026-01-21

**Authors:** Taraneh Westergerling, Vaneeda Allken, Webjørn Melle, Anne Gro Vea Salvanes, Shale Rosen

**Affiliations:** 1 Institute of Marine Research, Bergen, Norway; 2 Department of Biological Sciences, University of Bergen, Bergen, Norway; Central Marine Fisheries Research Institute, INDIA

## Abstract

Mesopelagic organisms play an important role in the ocean’s carbon transport and food webs and have been regarded as a potential harvestable resource. Their extensive aggregations in the upper thousand meters of the water column are frequently detected acoustically as deep scattering layers. However, extracting species and length composition from acoustics alone is challenging. Trawl catches, commonly used for ground-truthing acoustic data, suffer from size- and species-specific escapement and are spatially integrated along the trawl path. In-trawl cameras offer records at a finer spatial scale and are unaffected by mesh selectivity in the codend. Hence, integrating optical systems into trawling operations can enhance the validation of acoustic data without increasing sampling time. In this study, we trained a deep learning-based object detection model (YOLO11s) to automate the identification of seven mesopelagic groups common in the North Atlantic Ocean (lanternfish, silvery lightfish, barracudina, krill, pelagic shrimp, gelatinous zooplankton, and squid) along with a group of larger pelagic fishes from in-trawl images collected under white, and red-light with two gain settings. The model generally performed better on white-light images (weighted mean average precision ~ 0.95). However, using red light did not greatly reduce the model’s ability to detect mesopelagic organisms (weighted mean average precision ~ 0.77). The model performed especially well at detecting lanternfish, silvery lightfish and barracudina (average precision > 0.89). Object classes with average precision values under 0.80 (e.g., pelagic shrimp, krill) benefited from increasing the image resolution and expanding the training dataset. Our study demonstrates that employing the latest machine learning algorithms enables the detection of small-sized mesopelagic species from in-trawl camera images, allowing for rapid extraction of depth-stratified data and records of fragile species that are typically lost in the codend meshes.

## 1. Introduction

Between the euphotic zone (upper 200 m) and the depths of perpetual darkness (below 1000 m) lies the ocean’s twilight, or mesopelagic zone [1]. This zone encompasses approximately 20% of the global ocean’s volume [2] and is the habitat for a diverse group of organisms, many of which reside at depth to hide from visual predators during daytime and ascend to feed near the surface at night [3]. This diel vertical migration (DVM) links surface waters and the ocean deep, and contributes to carbon transportation [4]. Furthermore, mesopelagic organisms have been considered a potential harvestable resource [5,6] and play an important role in the ocean’s food webs by feeding on detritus and epipelagic zooplankton while being preyed upon by commercially targeted fish species, mammals, and pelagic squid [7].

Echosounders are less intrusive than nets and can sample on considerably larger and finer temporal and spatial scales [8–13]. This makes them ideal for detecting and observing the behaviour of mesopelagic animals, which can be densely or loosely aggregated across several hundreds of meters vertically and thousands of kilometres horizontally [2]. However, applying acoustics to estimate biomass or species composition is challenging [14,15]. Acoustic backscatter energy varies due to the presence or absence of gas-filled structures (e.g., swim bladders in fish), size, body orientation, and depth [8,14,16–18]. Given the low signal-to-noise ratio at depth for high-frequency echosounders, acoustic target classification of mesopelagic organisms during the day is limited to the lower frequencies [19].

In fisheries surveys, hull-mounted acoustics are typically applied in combination with net sampling techniques [12]. Trawl catches can provide precise taxonomic identification, length distribution and other population parameters such as age and sex [12]. The disadvantage of using trawls is that they spatially integrate over larger volumes than acoustic measurements [20,21], organisms can avoid the trawl opening [22,23], and fragile or small organisms are either destroyed beyond recognition or lost as they pass through the meshes [6,14,24]. Hence, ground truthing that relies solely on trawl catches will bias biomass indices for certain sizes and species and lose information on fine-scale spatial distribution.

To improve catch information in commercial fisheries and advance scientific trawl sampling, stereo-optical systems have been developed in the last two decades to identify, length measure, and count organisms as they move through the net [21,25–29]. When optical systems are placed ahead of the codend, more small and fragile organisms can be registered than are commonly found in the catch [6,26,30]. This shows the potential of in-trawl cameras to ground-truth acoustics on a much finer spatial and temporal resolution and for a wide range of organisms without increasing sampling time.

Manual analysis of in-trawl camera data is time-consuming [26,30]. To improve efficiency, researchers have started to rely on machine learning methods to extract the identification, length, and count of organisms from images or video [29,31–38]. So far, most networks trained on in-trawl images focus on identifying and counting commercial fish species [33,36,39], not fully utilising the camera’s ability to image small and fragile organisms.

Small objects present a greater challenge for object detectors compared to medium and large targets [40–42]. In a previous study, a neural network trained to detect larger pelagic fish was extended to include *Maurolicus muelleri*, *Benthosema glaciale*, and other common myctophids in the Norwegian Sea as a grouped class. However, this approach resulted in the model failing to detect over half of the mesopelagic fishes present [43]. This limitation likely reflects the small size of the available training dataset. Deep learning models typically require large amounts of annotated training data to learn key morphological features essential for automatic identification [44]. However, acquiring such data is often constrained by the scarcity of annotated data, as the annotation process is both labour-intensive and time-consuming.

Light is an important driver for the daily migratory behaviour and the global distribution of mesopelagic organisms [45–50]. Optical systems rely on artificial light to sample the water column where ambient light levels are insufficient, potentially affecting the natural behaviour of the surrounding organisms. When mounted on fishing gear, artificial lights have been shown to influence selectivity, leading to biases in species composition [51]. In addition to intensity, wavelength may also affect the reactions of organisms to artificial light sources. The eyes of most deep-sea fishes have low chromatic sensitivity, showing peak detection at shorter wavelengths, particularly in the blue and green ranges [52–54]. Recent work on mesopelagic fishes showed avoidance behaviour when exposed to white but not red artificial light [55,56]. This suggests that the use of white light for illumination may bias the species composition in “optical” samples, and the use of red light may be warranted.

The extensive vertical distribution of mesopelagic organisms makes depth-stratified samples from in-trawl cameras highly valuable. Therefore, this study exploits the potential of in-trawl cameras to sample small and fragile organisms and builds up a mesopelagic-focused object-detection model on images collected with white and red light (red light at two gain settings). To address the potential under-representation of specific object classes within the training data, we explored approaches to increase the size of the training dataset, including data augmentation strategies. Furthermore, we investigated the extent to which increased image resolution contributes to improved model performance, particularly with respect to the detection of small objects.

## 2. Materials and methods

### 2.1. Collection and processing

**Fig 1:**
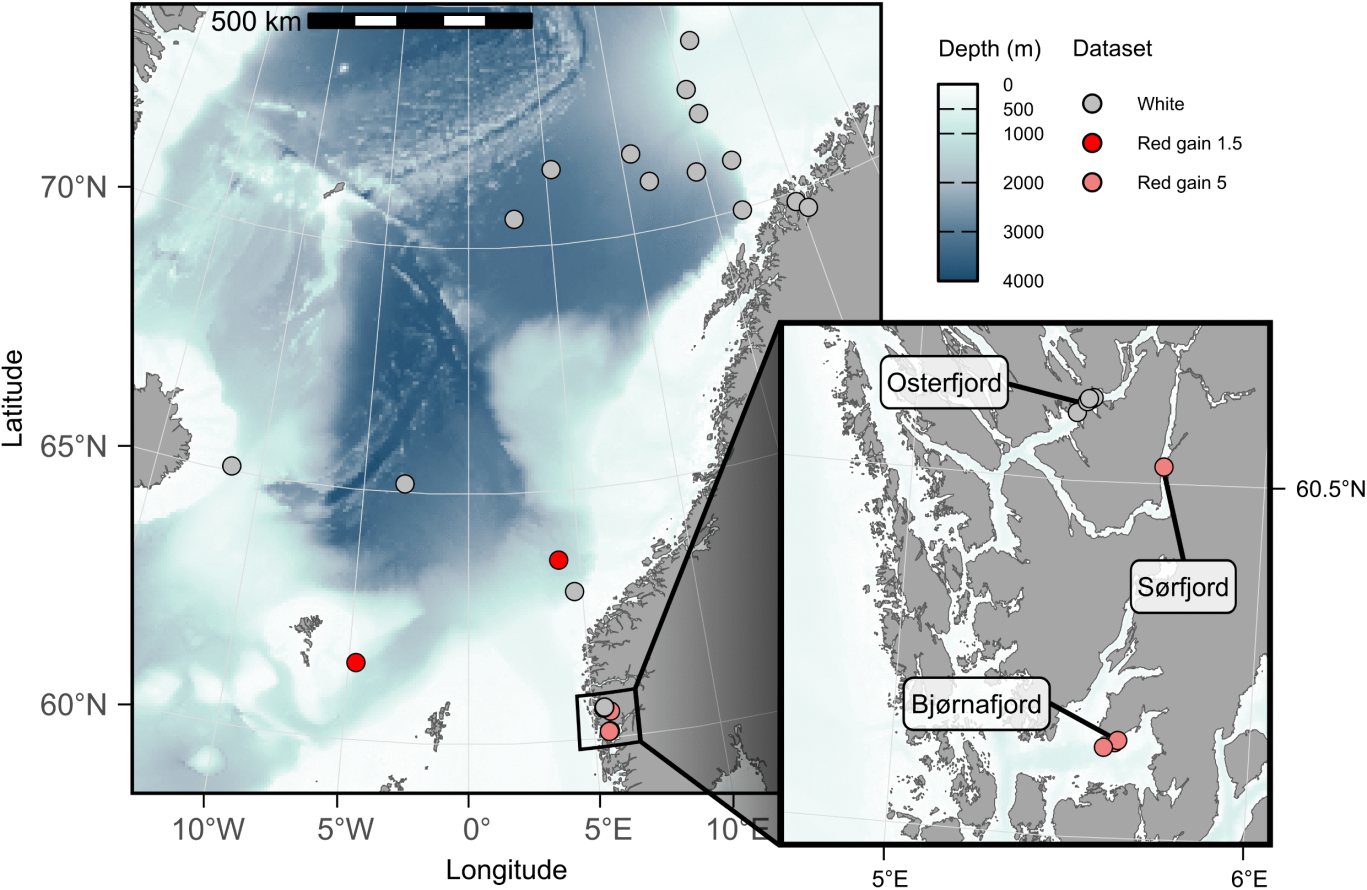
Study area. Map of the Norwegian Sea featuring locations of pelagic hauls (circles) used for the development of an object-detection model for mesopelagic organisms from in-trawl camera images. Circles are coloured based on image type, according to the lighting and camera gain used during collection: white, red gain 1.5, and red gain 5. Inset: Map of West-Norwegian fjords. The map was produced in R v. 4.5.2 [57], using the ggOceanMaps package v. 2.2.0 [58].

**Fig 2:**
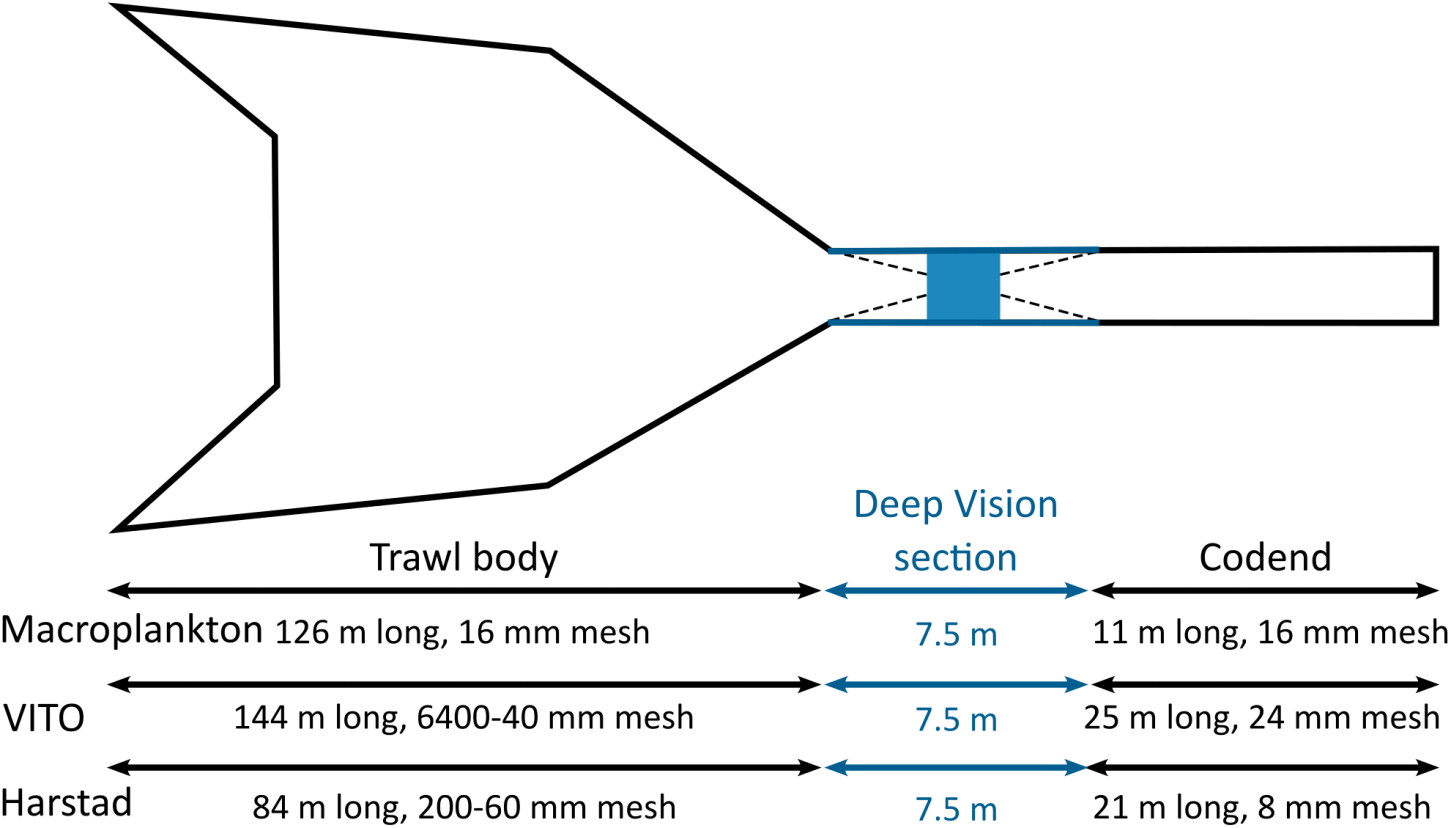
Mounting of the Deep Vision in-trawl camera system inside three models of pelagic trawls. The stretched circumference is 280, 397, and 320 m for the Macroplankton, VITO, and Harstad trawl, respectively. The Deep Vision section consists of two 3 m long sections that guide the organisms inside the imaging chamber of the 1.5 m long in-trawl camera system. The length and stretched mesh size are specified for the trawl body and codend of each of the three pelagic trawl models.

The data used in this study originate from a series of research surveys carried out in the Norwegian Sea, West-Norwegian fjords, and North-Norwegian fjords from 2020 to 2024 (Fig 1, Table 1). All images were collected using the Deep Vision camera system (Scantrol Deep Vision AS, Bergen, Norway), mounted between the extension and codend of one of three different pelagic trawls (Fig 2). The Deep Vision is described in greater detail in [59].

**Table 1:** In-trawl camera data included in this study. Period: sampling month and year; area: area the survey covered; trawl: types of pelagic trawls used ranging from macroplankton trawl to fish trawls (VITO, Harstad); gain: gain of the camera; frames per second: frame-rate at which images were recorded; resolution: image width and height in pixels; light: colour of the artificial light source.

Period	Area	Trawl	Frames per second	Resolution	Light and gain
March 2020	West-Norwegian fjords	Macroplankton	5	2456 × 2054	Red 5
June 2021	Norwegian Sea	Macroplankton	10	2456 × 2054	Red 1.5
February 2022	West-Norwegian fjords	Harstad	10	2456 × 2054	White 1.5
May 2022	Norwegian Sea	VITO	10	1228 × 1027	White 1.5
February 2023	West-Norwegian fjords	Harstad	10	2456 × 2054	White 1.5
May 2023	Norwegian Sea	VITO	10	1228 × 1027	White 1.5
November 2023	North-Norwegian fjords	VITO	10	1228 × 1027	White 1.5
February 2024	Norwegian Sea	VITO	10	1228 × 1027	White 1.5

**Fig 3:**
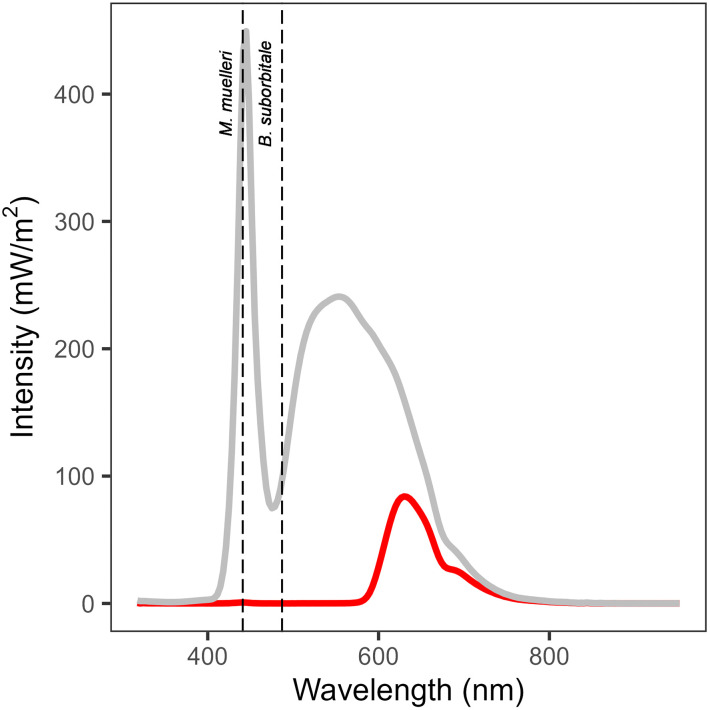
Intensity of light emitted by the Deep Vision in-trawl camera across wavelengths with white (grey curve) and red light (red curve). Dashed lines indicate peak sensitivity of Maurolicus muelleri (λmax 441 nm; see de Busserolles [53]) and Benthosema suborbitale (λmax 487 nm, see Douglas and Patridge [60]). Based on visual properties, B. suborbitale is the nearest relative to the dominant lanternfish B. glaciale for which light sensitivity data were available (see Fig 15 in de Busserolles [52]). The figure was produced in R v. 4.5.2 [57].

**Fig 4:**
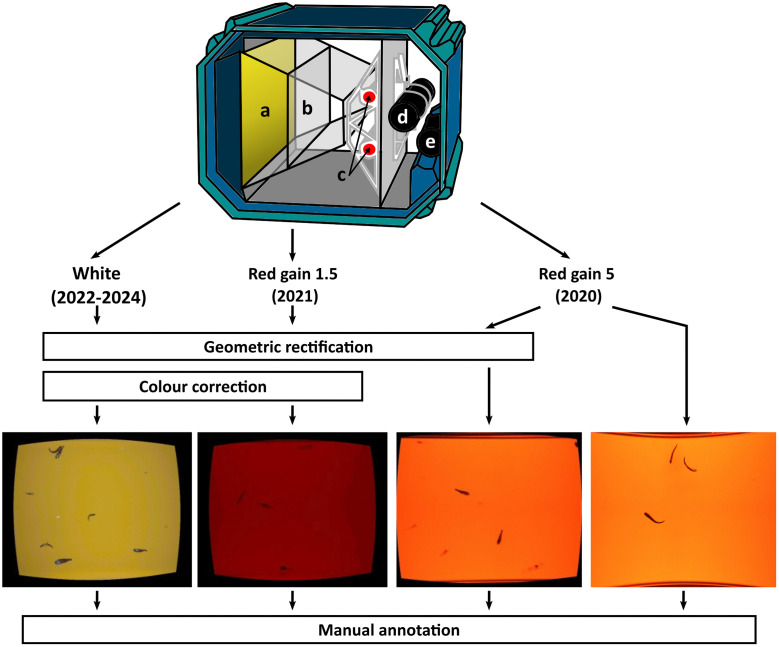
Detailed anatomy of the Deep Vision during 2022–2024, 2021 and 2020: (a) imaging chamber, (b) divider to reduce the volume of the imaging chamber (2021 only), (c) two stroboscopic lights, (d) stereo camera, and (e) battery. Image processing steps applied after collection using the “Deep Vision HMI” software (Scantrol Deep Vision AS, Bergen, Norway): geometric rectification to facilitate length measurements from images (final images with rounded sides); colour-correction applied to all images collected after the year 2020. Examples of the types of images used for manual annotation.

The data from surveys in 2022, 2023 and 2024 were all recorded using white light (peak output at 445 and 555 nm) (Fig 3, Fig 4). In 2020 and 2021, the surveys were dedicated to mesopelagic organisms. To prevent these organisms from perceiving the light from the camera system, a filter (peak transmission 630 nm, i.e., red light) was placed over the LED strobes (Fig 3). For the 2021 survey, the depth of the imaging chamber was halved to reduce the maximum range from the camera and aid in the identification of small objects (Fig 4). After collection, all images, except for a small number of red-light images from 2020, were geometrically rectified to facilitate the measurement of lengths from the images. From 2021 onward, an additional colour-correcting post-processing step was included, which led to the reduction of the camera’s gain from 5 to 1.5 during image collection, resulting in visibly darker red-light images. The geometric rectification and colour-correction were conducted using the “Deep Vision HMI” software (Scantrol Deep Vision AS, Bergen, Norway) prior to ML analysis and followed the procedures described in [34,61,62]. All the red-light images and half of the white-light images were collected at full resolution (2456 x 2054 pixels), while the other half of the white-light images were collected at half resolution (1228 x 1027) (Table 1). The various sampling designs, camera settings, and post-processing methods employed throughout this study’s timeline are a result of the ongoing development of the deep vision in-trawl camera system.

### 2.2. Light measurement

To test whether placing a filter over the stroboscopic lights would remove wavelengths corresponding to the peak sensitivity of the two most common mesopelagic fish species in the region (*M. muelleri, B. glaciale*), light intensity was measured at 320–950 nm wavelengths within the imaging chamber of the in-trawl camera using a spectral imaging radiometer (TriOS RAMSES ACC, TriOS Mess- und Datentechnik GmbH, Rastede) (Fig 3). Measurements were taken in air behind light-absorbing darkroom curtains, with the strobe lights of the in-trawl camera set to 5 Hz and an integration time of 8192 ms. Sixty-four measurements were collected over 604 seconds (8192 ms of integration plus 1246 ms for the radiometer to reset between measurement sequences). To compensate for any ambient light, measurements were also made over a 604 second interval with the camera and strobes off and subtracted from the measurements taken when the system was running. Since the sensor was placed just 90 cm from the light source, it was assumed that light absorption in water would be minimal over this range, and the spectrum in water would resemble that measured in air. Without the filter, the white light emitted from the in-trawl camera strobes included wavelengths matching the peak sensitivity of *M. muelleri* and *B. suborbitale* (λmax 441 and 487 nm, respectively; [53,60]) (Fig 3). The filter removed most wavelengths below 600 nm, effectively avoiding the peak sensitivities of these two mesopelagic fishes.

### 2.3. Building a mesopelagic detector

To automate the detection of mesopelagic organisms from in-trawl camera data, we employed the YOLO11s deep learning object detector [63], a machine learning algorithm designed to identify and locate objects within images. Benchmark studies have shown that recent versions of the *You Only Look Once* family of real-time object detection models outperform most single-stage object detectors like EfficientDet and RetinaNet both in speed and accuracy [64,65]. YOLO11 is one of the latest iterations of the YOLO family, incorporating architectural refinements such as the C3k2 backbone module for efficient feature extraction, Spatial Pyramid Pooling-Fast (SPPF) layer for robust multi-scale representation, and the C2PSA spatial attention mechanism to enhance fine-grained feature preservation. These innovations make this model particularly well-suited to the challenge of capturing morphological features of small objects without substantially increasing inference time. The YOLO11s-variant offers a balanced trade-off between performance and computational cost, as demonstrated by benchmark tests on the COCO dataset [63] making it a robust choice for both large-scale survey analysis and potential real-time field deployment of marine monitoring systems. To validate this choice, we compared the performance of five YOLO variants (S1 Fig, S2 Table). The models YOLOv9c, YOLO11s and YOLO11l demonstrated the highest performances, with comparable mean average precision scores. Among these, YOLO11s was selected for further use, due to its shorter training time, approximately three times faster than the other two top-performing models, making it a more efficient choice for this study, where we experiment with multiple models.

The model-building process involved four key steps: annotation, training, validation, and testing. First, a collection of representative images was manually annotated by identifying the category of each object within an image and noting its position using bounding boxes. The manually annotated datasets of each image type (white, red gain 5, red gain 1.5) were then split into training, validation, and test sets. To expand the training data without generating more manual annotations we projected existing annotations on images prior to colour correction or generated synthetic images by pasting crops of organisms on empty (“background”) images.

During the training phase, the model learned to recognise patterns associated with objects from different categories. It generated a score of vectors, one for each category and then measured the error between those scores and the desired pattern of scores, corresponding to the annotations [66]. It then adjusted its parameters to minimise this discrepancy and produced new outputs for a new batch of images from the training dataset. The model ran through the training set multiple times, with each complete pass referred to as an epoch. Over time, it reduced its error, developing the internal representation needed to identify objects in new images. In all experiments, we trained YOLO11s (pre-trained on the COCO dataset [67]) for 200 epochs, with an early stopping patience of 10 epochs. Unless otherwise specified in the experiments described below, default YOLO11s parameters were used (see S3 Table).

A separate validation dataset was used to evaluate the model’s performance periodically (after each epoch) during training. This helped monitor overfitting, i.e., ensured that the model’s performance generalised to unseen data. Mean average precision (mAP) was used as a performance metric to determine when the model had converged, so that the training could be stopped at the optimal point (Table 2).

**Table 2:** Acronyms and definitions of machine learning terms used throughout the manuscript. For a more detailed explanation of the performance evaluation metrics, refer to [68,69].

Acronym	Term	Definition
	Annotation	An object in an image that was either manually labelled or synthetically generated using the method described in [59]. All annotations are assumed to be true.
	Detection	An object in an image that was automatically detected by a machine learning algorithm.
TP	True positive detections	Objects that are detected automatically and have been annotated.
FP	False positive detections	Objects that are detected automatically but have not been annotated.
FN	False negative detections	Objects that are not detected automatically but have been annotated.
IoU	Intersection over union	$IoU=\;\frac{Area\;of\;Overlap\;between\;two\;bounding\;boxes\;}{Area\;of\;Union\;of\;two\;bounding\;boxes}$
	IoU threshold	Overlap between a detection and an annotation that must be met for the detection to be considered a TP.
	Confidence score	Score of how certain a model is that a detection belongs to a particular class.
	Confidence threshold	Confidence score that needs to be met for a detection to be considered TP.
	Confusion matrix	A matrix with the number of TP, FP, and FN for each class.
	Precision	Ratio of TP to total predicted positives: $Precision=\frac{TP}{TP+\;FP}$
	Recall	Ratio of TP to total actual positives: $Recall=\frac{TP}{TP+\;FN}$
	Precision-recall curve	Plot of precision and recall values at various confidence thresholds.
	F1-score	Harmonic mean of precision and recall: $F\text{1}=\frac{\text{2}}{\frac{\text{1}}{Precision}+\;\frac{\text{1}}{Recall}}$
	F1-confidence curve	Shows how the F1-score varies with the confidence threshold. The confidence threshold at which the F1 score is highest indicates the optimal confidence threshold for model prediction [31,70].
AP	Average precision	AP measures the area beneath the precision-recall curve, providing a single metric that indicates a model’s effectiveness in detecting each object class.
mAP	Mean average precision	Average of APs across all classes; used to assess overall model performance: $mAP=\;\frac{\text{1}}{c}\sum_{i=\text{1}}^{c}{AP}_{i}$ With c signifying the number of object classes and ${AP}_{i}$ the AP value for the object class i
Weighted mAP	Weighted mean average precision	mAP adjusted by class prevalence; useful for datasets with class imbalance: $weighted\;mAP=\;\frac{\sum_{c=\text{1}}^{n}\frac{{AP}_{c}}{a_{c}}}{\sum_{c=\text{1}}^{n}a_{c}}$ With n signifying the number of object classes,${\;a}_{c}$the number of annotations in the test set, and ${AP}_{c}$ the AP value for the object class c
NMS	Non-maximum suppression	Post-processing step to remove overlapping bounding boxes that predict the same object [71].
	NMS IoU threshold	Overlap between two automatic detections that is accepted before one is suppressed.

Once trained and optimised, the final performance of the machine learning model was evaluated on a set of new images (test set), using the performance evaluation metrics explained in section 2.4. Separate model runs, spanning training: validation and testing, were conducted with increasing training set size and image resolution to evaluate how these two factors influenced the model’s final performance.

#### 2.3.1. Manual annotation.

Stations likely to have high numbers of images containing mesopelagic organisms were identified through trawl catch records. Around 200 images were randomly extracted from the thousands collected at each sampled layer, where depth and trawling speed were constant. At a later stage, images were selectively chosen to increase the number of annotations of species that were under-represented by the random extraction. Using the software “LabelImg” [72], each object was manually identified and surrounded with a bounding box.

The annotations were grouped into the following seven mesopelagic object classes (Table 3, S1 Table): three types of fishes: lanternfish (mostly *Benthosema glaciale*, although it may also include other myctophids found in the Norwegian sea), silvery lightfish (*Maurolicus muelleri*) and barracudina (predominantly *Arctozenus risso*); two types of crustaceans: krill (dominated by *Meganyctiphanes norvegica*) and pelagic shrimp (*Pasiphaea* spp., *Eusergestes arcticus)*; as well as gelatinous zooplankton (e.g., *Periphylla periphylla*, *Aurelia aurita,* siphonophores); and squid (e.g., *Gonatus* spp.). The larger fishes, which co-occurred with the smaller mesopelagic organisms, were grouped into a generic fish class consisting of capelin (*Mallotus villosus*), blue whiting (*Micromesistius poutassou*), herring (*Clupea harengus*), saithe (*Pollachius virens*), redfish (*Sebastes* spp.) and gadidae (e.g., *Gadus morhua*, *Pollachius pollachius*, *Pollachius virens*).

**Table 3:** Annotations per object class (species groups) for both manually annotated, non-colour corrected, and synthetic datasets. The manually annotated datasets include colour-corrected white (W), colour-corrected red gain 1.5 (R1.5), and red gain 5 (R5) images indicated in bold. For each manually annotated dataset, the total number and proportion of annotations, as well as the numbers of annotations used for training (tr), validation (va), and testing (te), are provided. The non-colour corrected white (Wn), non-colour corrected red gain 1.5 (R1.5n), and synthetic red gain 5 (R5s) images were used to augment the number of annotations during training and were always used in combination with manual annotations (W_tr_, R1.5_tr_, R5_tr_). The last row displays the total number of images annotated for each dataset.

Object class\Dataset	Manually annotated												Non-colour corrected (n)		Synthetic (s)
	W_tr_	W_va_	W_te_	W	R1.5_tr_	R1.5_va_	R1.5_te_	R1.5	R5_tr_	R5_va_	R5_te_	R5	Wn	R1.5n	R5s
Lanternfish	2469	990	1027	4486 (0.16)	120	73	64	257 (0.04)	358	72	78	508 (0.23)	2001	120	3129
Silvery lightfish	6535	2034	2046	10615 (0.38)	263	93	95	451 (0.08)	131	33	48	212 (0.10)	6395	263	2867
Barracudina	90	45	50	185 (<0.01)	0	0	0	0	0	0	0	0	13	0	0
Fish	1754	729	715	3198 (0.12)	0	0	0	0	8	4	5	17 (<0.01)	756	0	2988
Krill	3871	1462	1542	6875 (0.25)	3079	1046	1045	5170 (0.87)	199	80	90	369 (0.17)	2391	3079	0
Pelagic shrimp	170	96	97	363 (0.01	0	0	0	0	315	110	117	542 (0.24)	144	0	0
Gelatinous zooplankton	648	312	297	1257 (0.05)	14	12	18	44 (<0.01)	341	112	113	566 (0.26)	473	14	0
Squid	384	134	133	651 (0.02)	0	0	0	0	0	0	0	0	380	0	0
Number of Images				6440				309				991	2742	194	2 968

To ensure that the model could be trained, validated, and tested independently on white and red-light images collected with a gain of 1.5 and 5, each manually annotated dataset (W, R1.5, R5) was handled separately. The three datasets were divided into training (tr), validation (va) and test (te) sets (60:20:20) using stratified partitioning so that the proportion of each class remained roughly similar (W_tr_, R1.5_tr_, R5_tr_, W_va_, R1.5_va_, R5_va_, W_te_, R1.5_te_, R5_te_) (Table 3).

#### 2.3.2. Data augmentation.

Data augmentation is a method used to artificially expand the training dataset by generating modified versions of the existing data [73]. In this study, the training set was augmented by projecting manual annotations onto non-colour-corrected images and by generating synthetic images. The non-colour-corrected images refer to those prior to colour correction and were available for half the W dataset and the entire R1.5 dataset (Fig 4). The synthetic images were produced using cropped objects with different orientations pasted at random locations on empty (“background”) images from the R5 dataset, following the procedures described in [59]. The datasets contained a total of 2742 non-colour corrected white-light images (Wn), 194 non-colour corrected red-light images collected with a gain of 1.5 (R1.5n) and 2968 synthetic red-light images with a gain of 5 (R5s) (Table 3). Wn, R1.5n, and R5s were only used while training the object detection model, and always in combination with manual annotations (W_tr_, R1.5_tr_, R5_tr_).

#### 2.3.3. Training datasets.

To investigate whether expanding the training dataset could improve model performance, we trained the model using nine distinct datasets at the highest image resolution (1216 px). To begin with, the model was trained and validated separately on white, and red-light images with gains of 1.5 and 5, using only the manually annotated images for training (W_tr_, R1.5_tr_, R5_tr_) and validation (W_va_, R1.5_va_, R5_va_) (Table 4). Then, non-colour-corrected or synthetic images were added to the training data (Wn_tr_, R1.5n_tr_, R5s_tr_). Lastly, the model was trained and validated on a combination of white and red-light images, with and without the addition of non-colour-corrected and synthetic images (WR_tr_, WRn_tr_, WRns_tr_, WR_va_).

**Table 4:** Composition of the annotation dataset across training (tr), validation (va) and test (te). In total, this study tested nine different training sets of varying sizes (W_tr_, R1.5_tr_, R5_tr_, Wn_tr_, R1.5n_tr_, R5s_tr_, WR_tr_, WRn_tr_, WRns_tr_). Validation (W_va_, R1.5_va_, R5_va_, WR_va_) and test sets (W_te_, R1.5_te_, R5_te_) consisted of only manually annotated images. The number of manual, non-colour corrected, and synthetic annotations for each object class can be found in Table 3.

Dataset		Training									Validation				Test		
		W_tr_	R1.5_tr_	R5_tr_	Wn_tr_	R1.5n_tr_	R5s_tr_	WR_tr_	WRn_tr_	WRns_tr_	W_va_	R1.5_va_	R5_va_	WR_va_	W_te_	R1.5_te_	R5_te_
Manually annotated	W	W_tr_			W_tr_			W_tr_	W_tr_	W_tr_	W_va_			W_va_	W_te_		
	R1.5		R1.5_tr_			R1.5_tr_		R1.5_tr_	R1.5_tr_	R1.5_tr_		R1.5_va_		R1.5_va_		R1.5_te_	
	R5			R5_tr_			R5_tr_	R5_tr_	R5_tr_	R5_tr_			R5_va_	R5_va_			R5_te_
Non-colour corrected	Wn				Wn				Wn	Wn							
	R1.5n					R1.5n			R1.5n	R1.5n							
Synthetic	R5s						R5s			R5s							

#### 2.3.4. Image resolution.

In YOLO models, images are typically resized by default so that the largest dimension is 640 pixels. To test whether increasing image resolution could improve the model’s performance for small-sized objects, the model was run on a range of image resolutions (width × height: 640 × 535, 736 × 616, 832 × 696, 1024 × 856, 1120 × 937, 1216 × 1017 pixels) using the largest training set (WRns_tr_). Throughout the manuscript, image resolution will be expressed as image width.

#### 2.3.5. Light and camera gain.

To evaluate if the model performed differently on white or red-light images with two gain settings, each model run was tested on separate datasets (W_te_, R1.5_te_, R5_te_). Moreover, the best-performing model across the three test sets (training set: WRns_tr_, image width: 1216 px) was examined using a range of evaluation metrics (described in Section 2.4 and Table 2) to pinpoint the factors driving the variations in performance. This analysis focused on lanternfish, silvery lightfish, krill, pelagic shrimp, and gelatinous zooplankton, as they were found in at least two test sets and had sufficient annotations.

For the three worst-performing object classes, an additional error analysis was performed to investigate the causes behind the high number of false positive (FP) and false negative (FN) detections. First, up to 50 FPs and 50 FNs were randomly selected for each species and test set (W_te_, R1.5_te_, and R5_te_). Then each bounding box was labelled with the cause resulting in an FP or FN (described in Table 5), along with the visible morphological features of the imaged object, whether there was overlap with other objects, and the degree of contrast with the background (good, average, bad). For krill and pelagic shrimp, we distinguished between the following visible morphological features: eyes, head, abdomen, antennae, body excluding antennae, and body including antennae. For gelatinous zooplankton, we differentiated between pigmented, partially pigmented, and transparent.

**Table 5:** Definitions of causes for false positive (FP) and false negative (FN) detections used in the error analysis. Cropped images from each test set to illustrate these causes can be found in S6 Fig.

Cause	Definition	Type of error	Panel of S6 Fig
Mismatch	Positional mismatch between annotation and detection bounding boxes leading to a lower intersection over union (IoU) than the set threshold of 0.5.	FP/FN	A
Partially detected Object	Partial detection of an object in the image resulting in a smaller bounding box than the annotated one.	FP/FN	B
Missed annotation	Object missed during annotation.	FP	C
Missed detection	Object missed by the object-detection algorithm.	FN	D
Misclassified	Detection or annotation assigned to the wrong object class.	FP/FN	E
Unidentifiable object	Detection of an unidentifiable object or a background artefact.	FP	F
Detection around multiple objects	Detection surrounding multiple imaged objects, leading to a much bigger bounding box than the annotated one.	FP/FN	G
Duplicate detection	Duplicate detection boxes around the same object, which were not eliminated during non-maximum suppression (NMS), due to a lower IoU than the set NMS IoU threshold of 0.4 or because they belonged to different object classes.	FP	H

### 2.4. Performance evaluation metrics

Throughout the validation process, model performance was regularly assessed using mAP (Table 2). To evaluate how training data, image resolution, light conditions, and camera gain affected the model’s final performance, we calculated the weighted mAP and AP for each test set (W_te_, R1.5_te_, and R5_te_). In addition, we examined the F1-confidence curve and the confusion matrix of the best-performing model to understand the factors driving the performance differences between white and red-light images with two gain settings. Weighted mAP and F1-score were chosen as key metrics because they offer robust evaluation in contexts characterized by class imbalance. For a detailed explanation of the performance evaluation metrics, refer to Table 2 and [63,64].

Throughout testing, confidence threshold was set to 0.05 and the non-maximum suppression (NMS) IoU threshold was 0.4. The confidence and NMS IoU threshold were selected following an exploratory analysis (S2 Fig, S3 Fig). Aside from the parameters specified above, all others were set to the default values as specified by Ultralytics (S3 Table).

## 3. Results

**Fig 5:**
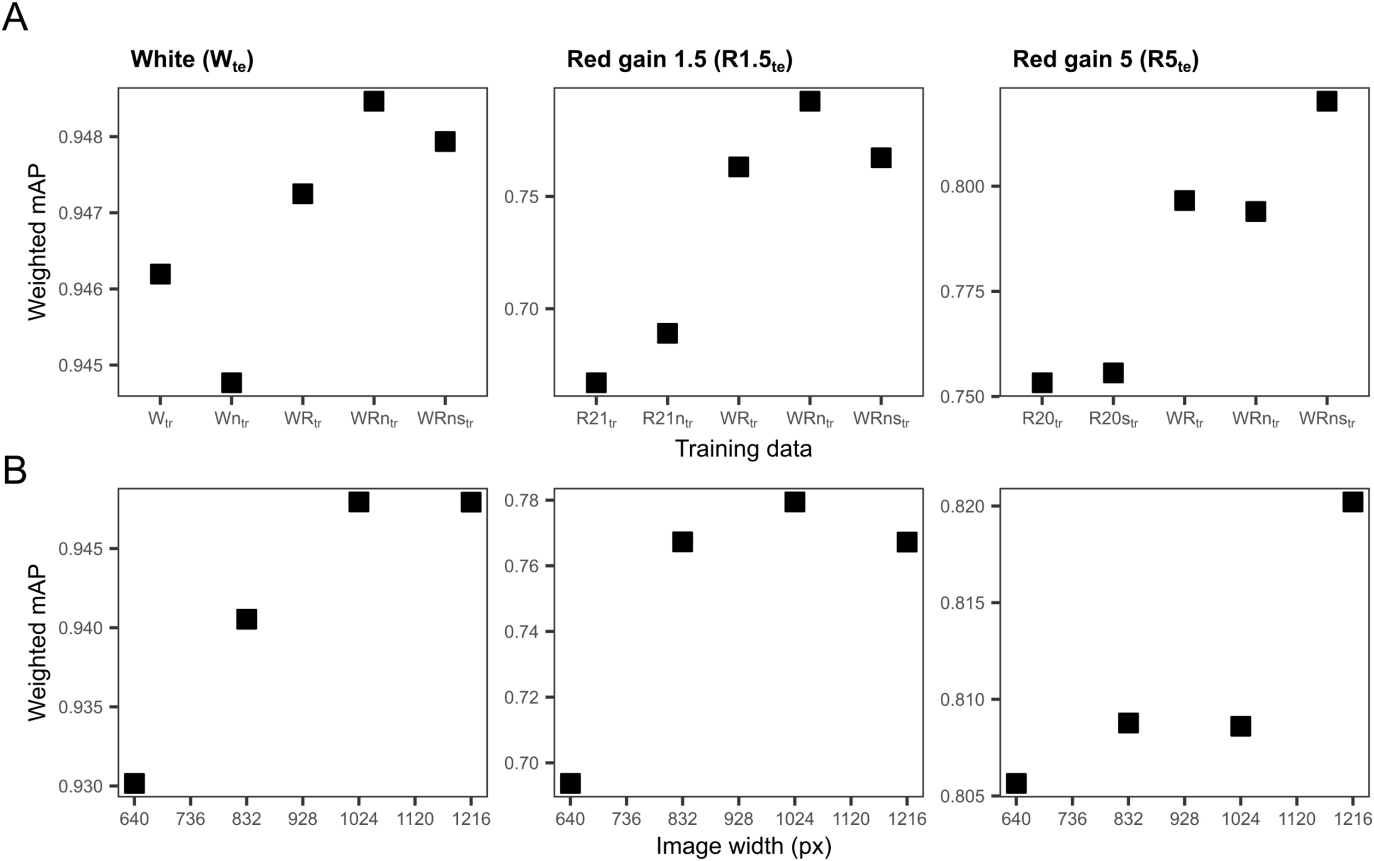
Effect of training set size and image resolution on weighted mean average precision (weighted mAP). (A) Effect of training set size on the weighted mAP of the mesopelagic detector at a constant image resolution of 1216 pixels. (B) Effect of image resolution (image width in pixels) on the weighted mAP of the mesopelagic detector for the model runs trained on WRns_tr_. The composition of each training set is described in Table 3 and Table 4. Performance was evaluated separately for each test set: white (W_te_), red gain 1.5 (R1.5_te_), red gain 5 (R5_te_). The figure was produced in R v. 4.5.2 [57].

**Fig 6:**
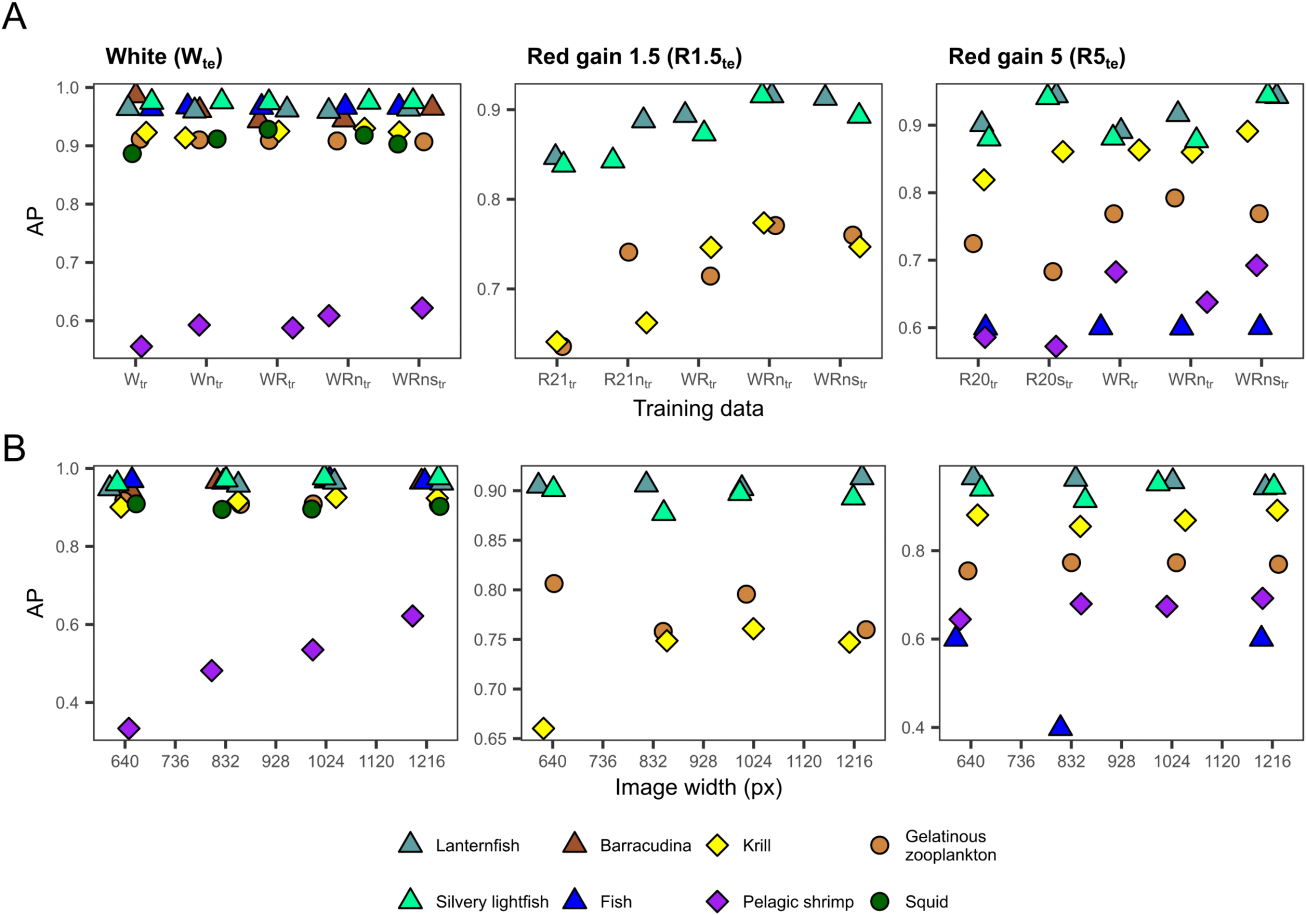
Effect of training set size and image resolution on average precision (AP) of each object class. (A) Effect of training set size on the AP of each object class at a constant image resolution of 1216 pixels. (B) Effect of image resolution (image width in pixels) on the AP of each object class for the model runs trained on WRns_tr_. Data points are coloured based on the object class and shaped according to higher taxonomic groups (triangle: fishes, diamond: crustaceans, circle: other). The composition of each training set is described in Table 3 and Table 4. Performance was evaluated separately for each test set: white (W_te_), red gain 1.5 (R1.5_te_), red gain 5 (R5_te_). The figure was produced in R v. 4.5.2 [57].

For most mesopelagic groups, the object detector performed well for all types of images, irrespective of the composition of the training set or image resolution. However, the overall performance (weighted mAP) was higher and less variable in white (W_te_: 0.93–0.95) compared to red gain 1.5 (R1.5_te_: 0.67–0.79) and red gain 5 (R5_te_: 0.75–0.82) images (Fig 5a, b). For white images, the model performed well on all the object classes (AP > 0.89) except for pelagic shrimp (Fig 6a, b). For red gain 1.5 and red gain 5, only lanternfishes and silvery lightfish maintained high APs (> 0.84). Gelatinous zooplankton and krill in red gain 1.5 images, and gelatinous zooplankton, pelagic shrimp and larger pelagic fishes in red gain 5 images tended to generally have AP values below 0.80.

### 3.1. Effect of training data

Models trained on a combination of white, red gain 1.5, and red gain 5 images (WR_tr_, WRn_tr_, WRns_tr_) generally performed better than those trained on only one type of image (W_tr_, R1.5_tr_, R5_tr_) (Fig 5a). A slight additional increase in weighted mAP was often noted when incorporating non-colour corrected and synthetic images (R1.5n_tr_, R5s_tr_, WRn_tr_, WRns_tr_). The red-light images benefited more from increasing the training set than white images, which generally displayed a higher weighted mAP > 0.92.

A closer look at the AP across object classes revealed that, in white-light images, pelagic shrimp was the primary group to benefit from the increased volume in training data (W_tr_: 0.56, WRns_tr_: 0.62) (Fig 6a). In red gain 1.5 images, all classes exhibited an increase in AP. However, krill and gelatinous zooplankton showed a higher rate of improvement (R1.5_tr_: 0.64, R1.5n_tr_: 0.77, for both object classes). In red gain 5 images, only krill (R5_tr_: 0.82, WRn_tr_: 0.89), gelatinous zooplankton (R5_tr_: 0.73, WRn_tr_: 0.79), and pelagic shrimp (R5_tr_: 0.59, WRns_tr_: 0.69) showed improvement in AP with more training data.

### 3.2. Effect of image resolution

The model’s weighted mAP increased with higher image resolution in white, red gain 1.5, and slightly in red gain 5 images (Fig 5b). In white images, pelagic shrimp was the only object class that benefited from increased image resolution (640 px: 0.33, 1216 px: 0.62) (Fig 6b). In red gain 1.5 images, solely krill showed an increase in AP with higher resolution (640 px: 0.66, 1024 px: 0.76), while no classes exhibited any improvement in red gain 5 images.

**Fig 7:**
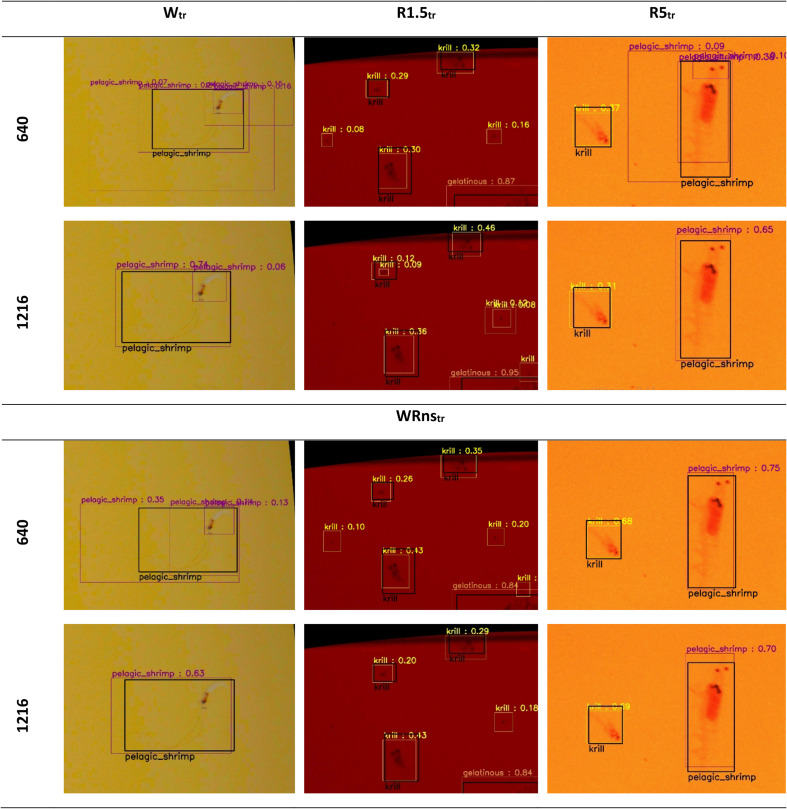
Cropped images from each test set to illustrate the effect of training set size and image resolution: white (W_te_, left column), red gain 1.5 (R1.5te, middle column), and red gain 5 (R5_te_, right column). Images are overlain with manual annotations (black) and automatic detections from model runs with varying training set size and image resolutions (coloured and labelled by object class). Columns: smallest training sets (W_tr_, R1.5_tr_, R5_tr_) and largest training set (WRns_tr_). Rows: minimum (640 pixels) and maximum (1216 pixels) image resolution. The composition of each training set is described in Table 3 and Table 4.

Review of the detection boxes drawn by the model revealed that for white images with low resolution (Fig 7), several detection boxes with varying confidence scores were often drawn around the same pelagic shrimp. These duplicate detections decreased when image resolution and training set size were increased.

### 3.3. Effect of light and camera gain

#### 3.3.1. Weighted mAP and AP.

**Fig 8:**
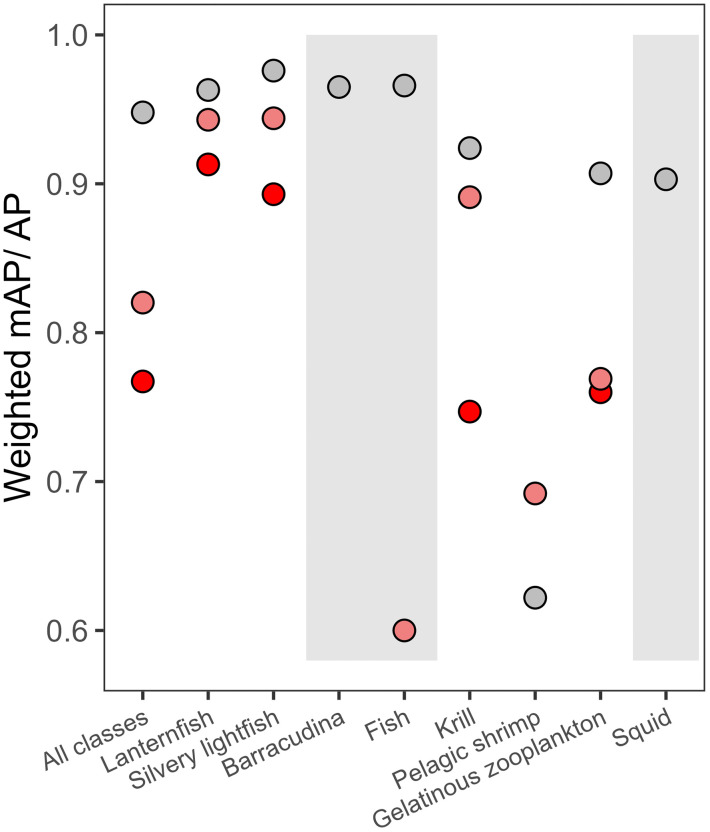
Weighted mean Average Precision (weighted mAP) and Average Precision (AP) of each object class for the best-performing model (training set: WRns_tr_, image width: 1216 pixels). The model was tested separately on white (W_te_, grey), red gain 1.5 (R1.5_te_, dark red) or red gain 5 (R5_te_, light red). The training and test sets are described in Table 3 and Table 4. The AP of all object classes were used to calculate the mAP. However, the object classes with a grey background were excluded from the analysis since they were present in only one of the test sets or had too few annotations. The figure was produced in R v. 4.5.2 [57].

The weighted mAP of the best-performing model (training set: WRns_tr_, image width: 1216 pixels) was highest for white-light images (0.95), followed by red gain 5 (0.82) and red gain 1.5 images (0.77) (Fig 8). For lanternfish and silvery lightfish, AP was generally high and consistent across different image types (> 0.89). For krill, AP was high in white (0.92) and red gain 5 (0.89) images, but was notably lower in red gain 1.5 images (0.75). Pelagic shrimp exhibited the lowest AP values of all the object classes. Additionally, it was the only object class where AP was greater in red gain 5 (0.69) compared to white images (0.62). For gelatinous zooplankton, AP in white images was 0.90, while red images, had lower AP values of 0.77 and 0.76 for gain 1.5 and gain 5, respectively. Barracudina, larger pelagic fishes, and squid were not included in the comparison as they lacked sufficient annotations cross lighting types (Table 3).

#### 3.3.2. F1-confidence curve.

A high F1 score reflects a model’s ability to effectively minimize both false positives and false negatives, thereby indicating a well-balanced trade-off between precision and recall. The confidence threshold which yields the highest F1-score has previously been used to determine the optimal threshold for model prediction [31,70].

**Fig 9:**
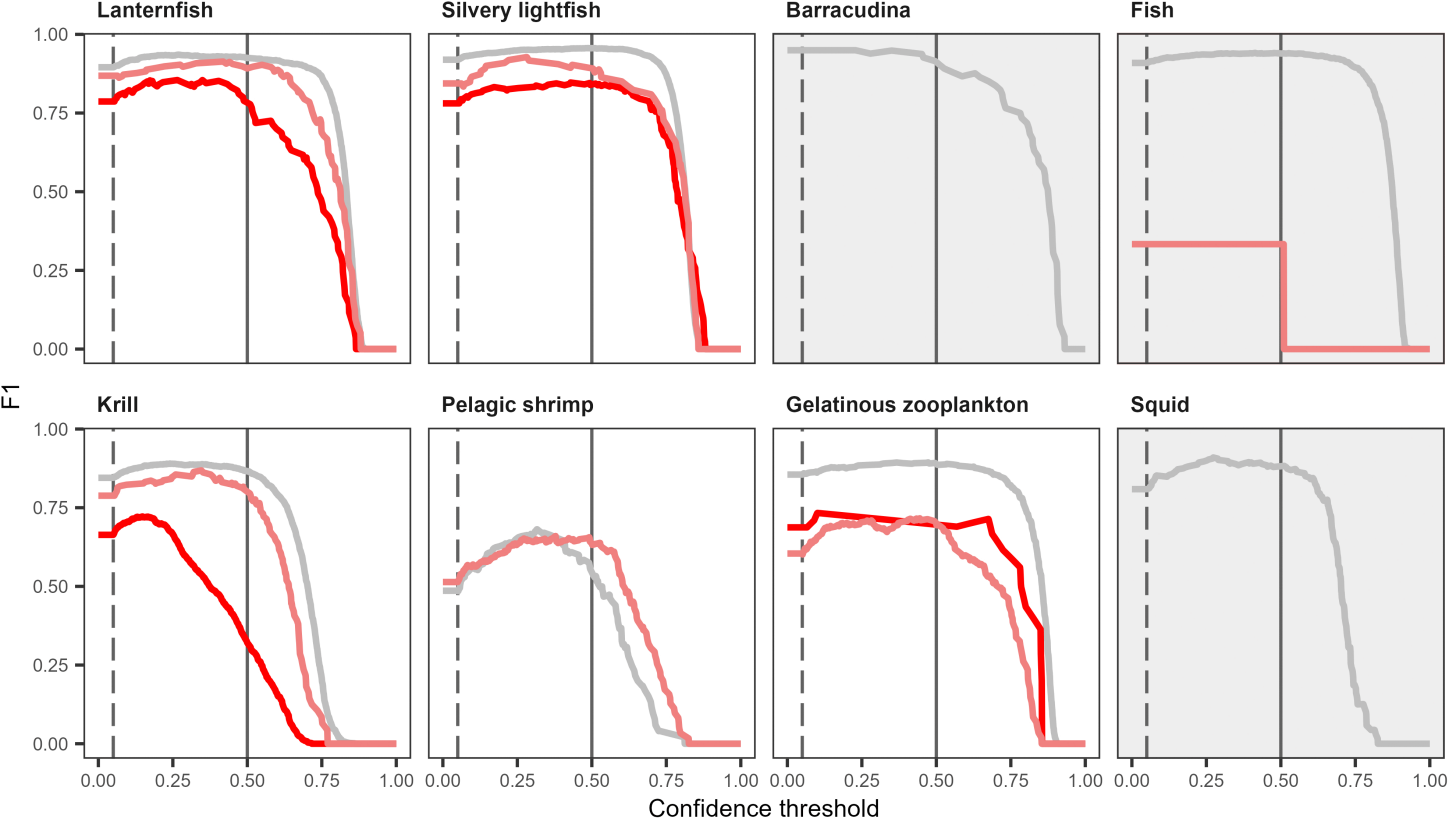
F1-confidence curves of each object class for the best-performing model (training set: WRns_tr_, image width: 1216 pixels). The model was tested separately on white (W_te_, grey), red gain 1.5 (R1.5_te_, dark red) or red gain 5 (R5_te_, light red). The dashed line represents a confidence threshold of 0.05, which was the value used at evaluation. The solid line represents a confidence threshold of 0.5 and serves as a reference point. The object classes with a grey background were excluded from the analysis since they were present in only one of the test sets or had too few annotations. The figure was produced in R v. 4.5.2 [57].

For the majority of object classes and image types, the F1-score remained relatively stable across confidence thresholds ranging from 0 to 0.5. An exception was noted for krill in the red gain 1.5 test dataset, where the F1-score started to decline already at a confidence score of 0.25 (Fig 9). Moreover, the F1-confidence curve for gelatinous zooplankton in the same test set, represented by only 18 annotations, exhibited greater variability and less smoothness compared to curves for other object classes, which had a minimum of 48 annotations.

#### 3.3.3. Confusion matrix.

**Fig 10:**
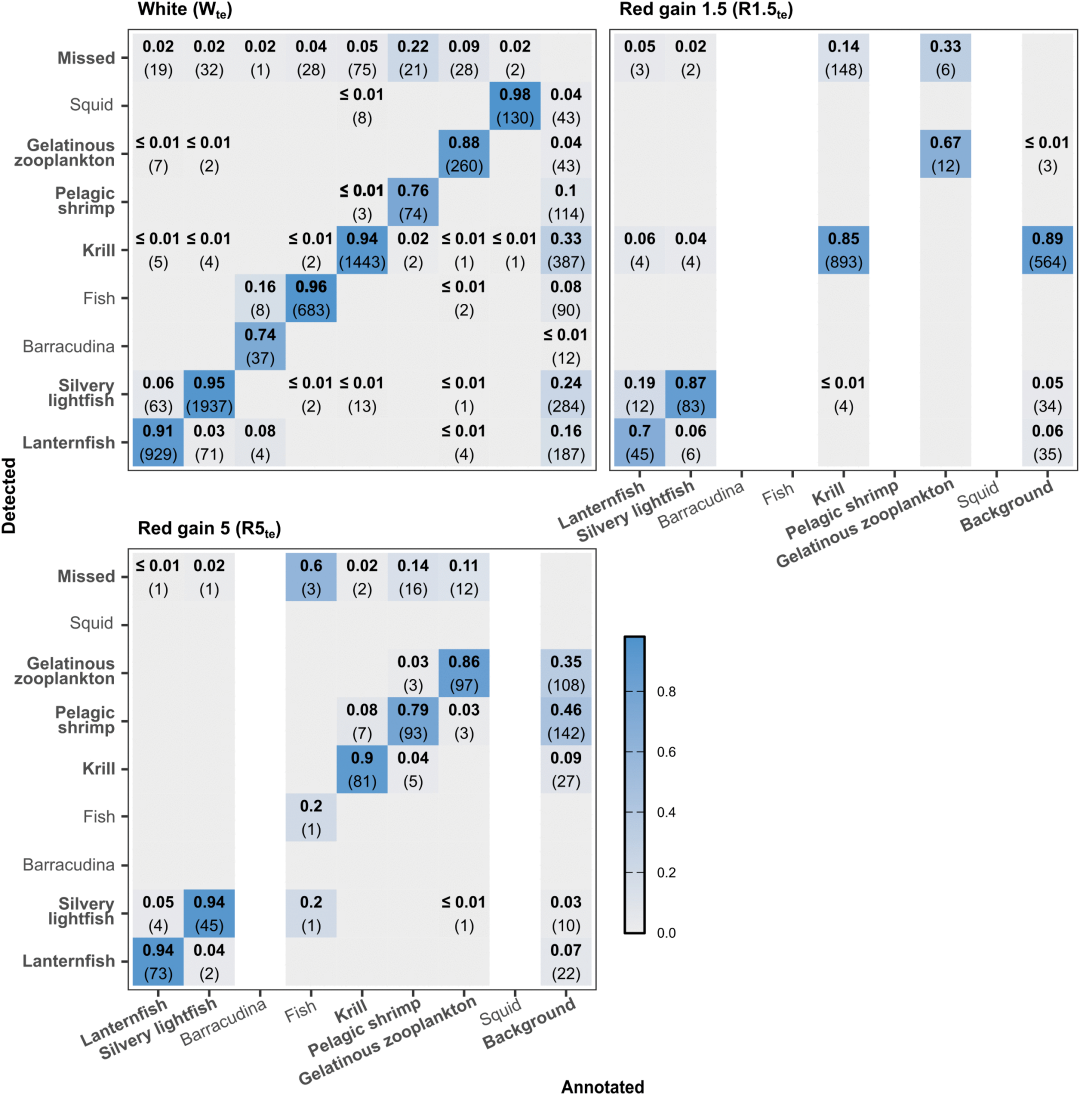
Confusion matrices of the best-performing model (training set: WRns_tr_, image width: 1216 pixels). The matrices were generated at the default confidence threshold of 0.25 and IoU threshold of 0.5 for each test set: white (W_te_), red gain 1.5 (R1.5_te_), red gain 5 (R5_te_). Object classes in bold are the focus of this analysis, as they were present in at least two test datasets and had sufficient annotations. Missed: annotations in the test set that the model did not detect. Background: model detections that were not annotated. The figure was produced in R v. 4.5.2 [57].

In the confusion matrix, “background” detections refer to false positive (FP) detections of objects that have not been annotated (Table 2, S6 Fig, Fig 10). For all three types of images, krill and pelagic shrimp had more background detections than missed or misclassified detections (Fig 10). In red gain 1.5 images, 89% of the background detections were attributed to krill. In red gain 5 images, nearly half (46%) of the background detections were identified as pelagic shrimp.

#### 3.3.4. Error analysis.

**Fig 11:**
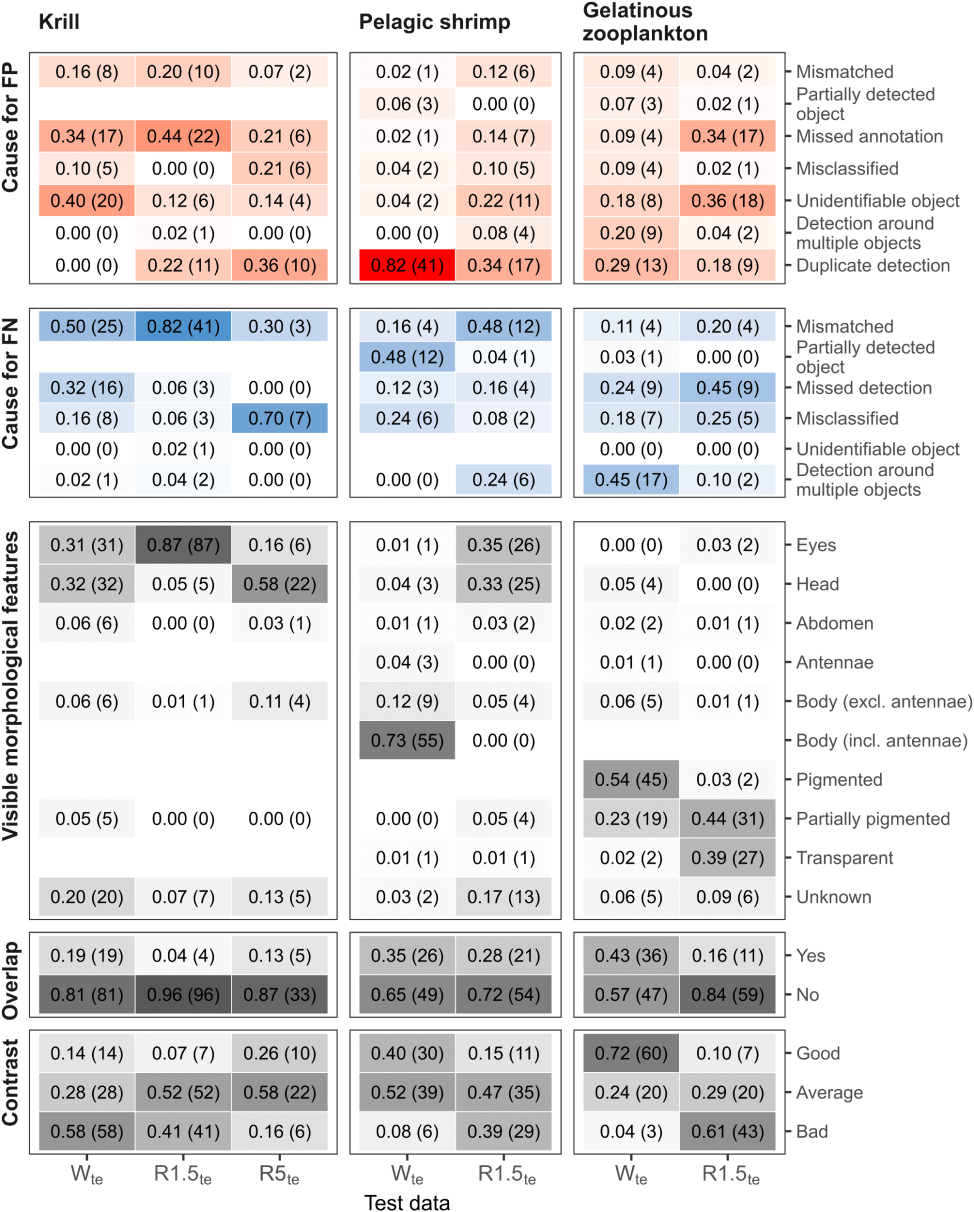
Analysis of false positive (FP) and false negative (FN) detections of krill, pelagic shrimp, and gelatinous zooplankton for the three test sets: white (W_te_), red gain 1.5 (R1.5_te_), red gain 5 (R5_te_). The detections were produced by running the best-performing model (training set: WRns_tr_, image width: 1216 pixels) and applying a confidence threshold of 0.05 and an IoU threshold of 0.5 on the images of the three test sets. Annotations are human labels assigned to each object in an image. Up to 50 FPs and FNs were randomly subsampled from each test set. For each FP or FN bounding box, the immediate cause of the error was noted, as well as its visible morphological features, overlap with other objects, and the degree of contrast with the background. Values are provided in proportions, and the number of bounding boxes is in parentheses. For krill in the R5_te_ test set, only 10 FN annotations were available; therefore, the proportions should be treated with caution. Due to misclassification, some visible morphological features for krill and pelagic shrimp include pigmented, partially pigmented, and transparent, which were only used for gelatinous zooplankton and vice versa. The figure was produced in R v. 4.5.2 [57].

A notable proportion of false positive (FP) detections occurred on objects that were overlooked during annotation. This was particularly evident for krill across all image types (W_te_: 34%, R1.5_te_: 44%, R5_te_: 21%), pelagic shrimp in red gain 5 (14%), and gelatinous zooplankton in red gain 5 (34%) (Fig 11). For krill in white images and gelatinous zooplankton in red gain 5 images, most FPs were objects that a human could not accurately identify or distinguish from background artefacts (40% and 36%, respectively). Except for krill in white images, many FPs were duplicates, especially for pelagic shrimp in white images (82%). However, it is important to note that for all three object classes more than half of the FP detections have confidence scores below the optimal threshold identified by the F1-confidence curve. (W_te_: 0.326, R1.5_te_: 0.164, R5_te_: 0.27) (S7 Fig).

For small objects, even a minor positional mismatch between detection and annotation bounding boxes can result in IoUs below the set threshold (< 0.5). For krill in white and red gain 1.5 images and pelagic shrimp in red gain 5 images, this was the primary reason for FNs (50%, 82%, 48%, respectively) (Fig 11). For pelagic shrimp in white images, it was the partial detection of an object that caused most of the FNs (48%). In white images, most FNs for gelatinous zooplankton resulted from detections around multiple objects in the images. In contrast, 45% of gelatinous zooplankton in red gain 5 images were genuinely missed.

The morphological features that were visible in the FP detections and FN annotations depended on the object class and image type. Among the errors analysed (i.e., false positives and false negatives combined), 87% of the krill in the red gain 1.5 image set were characterised by being visible only as pairs of black eyes. In white and red gain 5 images, the entire heads were visible 32% and 58% of the time, respectively. For pelagic shrimp, the body, including the two long antennae, was only visible under white light (73%). Under red light with a gain of 5, only the head (33%) or eyes (35%) of most pelagic shrimp were visible. Partially pigmented gelatinous zooplankton appeared in both the white and red gain 5 images (23% and 44%, respectively). However, pigmented gelatinous zooplankton were only present in the white images (54%), while transparent ones were found only in the red gain 5 images (39%).

The overlap of objects primarily occurred for gelatinous zooplankton in white images (43%) and to a lesser extent for pelagic shrimp (W_te_: 35, R5_te_: 28%) (Fig 11). The contrast with the background was either bad or average for most FP detections and FN annotations. However, for krill in red gain 5 pelagic shrimp and gelatinous zooplankton in white images, even objects with good contrast were labelled as FPs or FNs (26%, 40%, 72% respectively) (Fig 11).

## 4. Discussion

To harness the capacity of in-trawl cameras for obtaining depth-stratified samples of small and fragile organisms, we trained a machine learning model to detect seven mesopelagic groups along with larger pelagic fishes commonly encountered in the North Atlantic Ocean. The overall results suggest that while the model performed well in detecting mesopelagic fishes, its performance on other object classes was more variable depending on the training set, image resolution, light, and camera gain used during collection.

### 4.1. Mesopelagic fishes

Model performance on mesopelagic fishes remained consistently high with an average precision greater than 0.77, regardless of the training set, image resolution, light or camera gain. Pena [55] and Underwood [56] observed avoidance behaviour of mesopelagic fish when exposed to white but not red light. Therefore, species composition in samples collected via in-trawl cameras may underestimate these species when using white light for illumination. The model’s consistently high performance on mesopelagic fishes suggests that utilising red light to prevent avoidance behaviour does not hinder our ability to detect these organisms automatically. Compared to other object classes, mesopelagic fishes have a defined shape without elongated appendages, potentially making them easier to detect regardless of image resolution.

### 4.2. Crustaceans

In white-light and red-light images captured at a gain of 5, krill exhibited only marginally lower detection rates than mesopelagic fishes, regardless of training set or image resolution. In contrast, the model performed notably worse at detecting krill in the relatively darker red-light images that were recorded at a gain of 1.5. This improved slightly when increasing the size of the training set and image resolution.

Krill in white and red images recorded at a gain of 1.5 were markedly smaller than in red images at a gain of 5, with most bounding boxes having a diagonal smaller than 100 pixels (S5 Fig). The difficulty in detecting small objects can stem from fewer pixels being available for feature recognition, a higher likelihood of blending in with the background or overlapping objects, and low IoUs from slight mismatches between the placement of predicted and annotated bounding-boxes [40,74]. The lower average precision at 640 pixels compared to 832 pixels suggests that the standard resolution of the YOLO11s network is insufficient for accurate feature recognition of small krill in red images with a gain of 1.5. Furthermore, the rapid decline in the F1-score beyond a confidence threshold of 0.25 also indicates low confidence in many of the detection boxes for krill in red gain 1.5 images, confirming the higher uncertainty in detecting krill in this specific dataset (Fig 9).

Pelagic shrimp presented the greatest challenge for automated detection (AP < 0.70). Notably, it was the only class to perform better in red light images compared to white light. The pelagic shrimp object class is morphologically diverse, containing *Eusergestes arcticus* and multiple *Pasiphaea* species (S1 Table). This diversity may have hindered the models’ ability to learn the key features of the object class from the available annotations. The rise in performance with training set size, which continued to increase without plateauing, suggests that having more than 1000 annotations would likely further improve the detection of pelagic shrimp.

The lower performance for white compared to red images recorded at a gain of 5 for pelagic shrimp likely stems from the thin, elongated red antennae, which were annotated only under white light and were present in 73% of the FPs and FNs (Fig 11). These appendages require a high image resolution for detection, leading to duplicate detection boxes around the same object, increased spatial mismatch between predicted and annotated bounding boxes, and ultimately reduced overall performance (Fig 7, Fig 8, Fig 11, S6 Fig). Even when using the highest resolution of 1216 pixels, 82% of FP detections in white images were duplicates, and 48% of FN annotations were missed because detections only covered parts of the imaged object (e.g., body excluding antennae) (Fig 11). Although the long antennae aid in manually recognising pelagic shrimp, they may not be essential for automatic detection. A study that automated the detection and measurement of body length in Pacific white shrimp achieved an average precision of 93% without including antennae in the annotation [75]. Additionally, standard measurements for crustaceans such as carapace and total length do not include antennae [76].

### 4.3. Gelatinous zooplankton

Gelatinous zooplankton were detected well in white but not in red-light images. Like pelagic shrimp, gelatinous zooplankton encompass various taxonomic groups, including pigmented species such as *Periphylla periphylla* and *Cyanea capillata*, as well as mostly translucent *Aurelia aurita* and siphonophores. In the annotated dataset, white images featured only *Periphylla periphylla*, whereas the red-light images could contain translucent species (Fig 11, S1 Table). For translucent organisms, it is harder to obtain morphological information from underwater images due to their low contrast with the background and high-speed motion blur [77–79]. Moreover, translucent organisms are more likely to resemble background artefacts, which would explain the high rate of unidentifiable false positive detections (36%) and missed annotations (45%) in red images recorded at a gain of 5 (Fig 10, Fig 11). The even illumination and uniform yellow background of the Deep Vision imaging chamber reduces background interference and performs well for fish species, for which it is designed [26]. However, this setup may not be ideal for observing translucent organisms such as *A. aurita* and siphonophores. For example, the Scripps Plankton Camera system [80], uses darkfield illumination to enhance the edges of translucent organisms [81]. Future designs of in-trawl cameras could consider adapting the lighting or background to benefit the automatic detection of a wider range of species.

### 4.4. Dataset size and image resolution

The performance of deep learning models generally improves with the amount of labelled data, as more data allows models to learn the underlying patterns better and generalise to unseen data [44]. This may explain the overall lower performance in detecting mesopelagic organisms in the smaller red gain 1.5 (309 images) and red gain 5 (991 images) datasets compared to the white dataset (6440 images). The red gain 1.5 dataset benefited the most from an expanded training set. In the other two datasets, only classes with fewer annotations and more complex morphological features, such as pelagic shrimp in white, as well as krill, pelagic shrimp and gelatinous zooplankton in red gain 5 images, exhibited improved average precision with training set augmentation. This suggests that while some object classes (e.g., mesopelagic fish) are sufficiently represented in the training set, model performance for other classes (e.g., krill and gelatinous zooplankton under red light, and pelagic shrimp) could be enhanced through the inclusion of additional annotations. Future efforts to improve model performance could therefore involve a more targeted expansion of the dataset to address class imbalance [82].

K-fold cross-validation [83] could have improved model performance. However, in our study, many of the training sets contained augmented data that had to be excluded from validation folds, and others combined images collected under different lighting conditions. Proper stratification would have required balancing both species and lighting. These constraints made the use of k-fold incompatible with our experimental design.

The goal of automating the image analysis in this study has been to provide taxonomic information with greater spatial and temporal resolution than the catch to improve the scrutinisation of acoustic data during scientific surveys. This scrutinisation is typically conducted several hours after trawling and does not require real-time data extraction from optical systems. Accordingly, this study prioritised detection accuracy and the final model used the highest available resolution. However, real-time information becomes vital when applying open-closed codend systems to target only certain species or sizes, either during surveys or in commercial fisheries. In such scenarios, it may be necessary to prioritise inference speed over model accuracy. For instance, reducing image resolution can lower computational demands, thereby accelerating processing time, but potentially at the cost of reduced model performance [36,84].

### 4.5. Error analysis and model refinement

To improve model performance, it is essential to understand the underlying causes of errors. An analysis of false positives and false negatives points to three primary sources: model limitations, suboptimal inference parameters, or inaccuracies in the ground truth annotations. Identifying and categorising these sources enables targeted interventions that can enhance both model accuracy and reliability.

#### 4.5.1. Annotation quality.

Annotation errors are a common source of misclassification during manual review of images. Inconsistencies may arise when multiple annotators are involved or when annotation is conducted over extended periods. Factors such as annotator expertise and image quality further influence annotation accuracy, leading to missed or incorrectly labelled objects.

In this study, the transition from white to red-light imaging introduced challenges for manual annotation. Red light produces monochrome images, which can reduce object visibility and make it more difficult to recognise morphological features. This issue was exacerbated in 2021 when both LED strobe filters and reduced gain settings were used, resulting in darker images with lower contrast. These conditions increased the likelihood of missing small or translucent organisms. For example, 44% of false positive krill detections, visible only as black eyes in images recorded under red light and a gain of 1.5, and 34% of gelatinous zooplankton in red-light images with a gain of 5, were missed during manual annotation (Fig 11).

To mitigate these issues, red-light images could benefit from specialised image enhancement techniques [82]. Alternatively, a model trained on a subset of annotated data could be used to generate bounding boxes for the remaining images. These automatically generated boxes could then be reviewed by annotators, reducing the likelihood of missed detections and streamlining the annotation process.

#### 4.5.2. Inference parameter selection.

A substantial proportion of errors can be addressed by optimising inference parameters, particularly the Intersection over Union (IoU) and confidence score thresholds. It is important to distinguish between the IoU threshold used for evaluation and that used for non-max suppression (NMS). The evaluation IoU threshold determines whether a predicted bounding box sufficiently overlaps with a ground truth annotation to be considered a true positive. In contrast, the NMS IoU threshold governs the degree of overlap allowed between predicted boxes before they are considered redundant and suppressed.

For small objects, even minor positional deviations can result in a detection being classified as a false negative due to insufficient overlap with the ground truth (i.e., positional mismatch) [40,74]. Lowering the evaluation IoU threshold can increase tolerance for such deviations, improving model performance for small objects. Duplicate detections, on the other hand, can be reduced by lowering the NMS IoU threshold, which leads to more aggressive suppression of overlapping predictions. Additionally, increasing the confidence score threshold can help filter out low-quality detections of ambiguous or unidentifiable objects. Selecting optimal score thresholds, either globally or per class, can help balance false positives and false negatives, improving overall model performance.

#### 4.5.3. Real-life applications.

This analysis indicates that a substantial proportion of the observed errors, such as positional mismatches and missed annotations, are closely tied to the quality of the test set annotations. These issues are unlikely to affect model performance when applied to novel, unannotated images. After optimizing the model’s inference parameters, the primary remaining sources of error are expected to be misclassifications, duplicate detections (false positives), and cases where multiple adjacent objects are merged into a single detection, resulting in false negatives.

As discussed in earlier sections, targeted expansion of the training dataset can help address these issues. In particular, reducing false negatives caused by merged detections requires incorporating more densely populated scenes into the training data. This would enable the model to better distinguish between closely spaced or overlapping objects, thereby improving detection granularity and overall performance.

### 4.6. Ecological implications

Mesopelagic organisms hold both ecological [4,7] and commercial value in the oceans [5,6]. However, the absence of optimal sampling methods is a key factor contributing to knowledge gaps and uncertainties of global biomass estimates [5,14,85]. The integration of object detection models with acoustic data presents new opportunities to address these gaps. Siphonophores, a type of gas-bearing gelatinous zooplankton, are strong acoustic targets that have been identified as a major source of uncertainty in the biomass estimation of mesopelagic fish [14]. Due to their fragile nature, siphonophores are nearly impossible to sample using nets [86]. The model developed in this study could be used to provide depth-resolved data on the densities of these organisms and provide key information to improve biomass estimations. Across the water column, larger myctophids are generally found deeper than smaller ones [87,88]. Since acoustic backscatter of gas-bearing mesopelagic fish is a function of size and depth [18], future implementation of automatic sizing from images could provide depth-resolved data that could further improve its validation.

Moreover, long-term monitoring is generally focused on commercial species, which is also reflected in the prior efforts in developing object-detection networks for in-trawl images [25–29,35]. Since cameras are less size-selective than nets, trawl deployments designed to capture larger pelagic fish are likely to yield images containing smaller organisms even if they are not retained in the codend [6,30,31,43]. By providing a method to sample small and fragile organisms during existing surveys for commercial species, we could greatly expand the data on mesopelagic organisms, filling knowledge gaps on temporal changes in vertical and horizontal distributions, without requiring additional surveys or personnel.

### 4.8. Conclusion

Surveys targeting mesopelagic organisms, which can be densely or loosely aggregated across several hundred meters vertically, can greatly benefit from the temporal and spatial resolution of in-trawl cameras. The mesopelagic detector developed in this study enables rapid extraction of depth-stratified data on fragile species that are usually lost in the meshes of the codend. When considering only the overall performance of the model (mAP), it appears that the automatic detection of mesopelagic organisms is better when using white than red light. However, a closer examination of each object class revealed that mesopelagic fishes performed equally well irrespective of the light used during collection, and that pelagic shrimp performed better under red light. Small krill and organisms with thin, elongated appendages, such as pelagic shrimp, benefit from increased image resolution. Translucent organisms and object classes containing several species require a higher number of annotations for the model to learn their distinct morphological characteristics. Employing red light to minimise avoidance behaviour and potential biases should, therefore, not affect the capacity to identify mesopelagic organisms automatically, provided that an adequate image resolution is chosen and sufficient annotations are available.

## Supporting information

S1 Table Number of manual annotations for pelagic shrimp, gelatinous zooplankton and fish based on the original labels. (PDF)

S1 Fig Experiments to test the effect of model architecture (YOLOv8n, v9c, 11s, 11n, 11l). (PDF)

S2 Table Comparisons of YOLO models, runtime, accuracy. (PDF)

S3 Table Model parameters used during training as specified by Ultralytics. (PDF)

S2 Fig Experiments to test the effect of confidence threshold (0.25, 0.05) on the performance of the mesopelagic. (PDF)

S3 Fig Experiments to test the effect of threshold intersection over union (IoU) (0.7, 0.6, 0.5, 0.4) during non-maximum suppression on the best-performing model. (PDF)

S4 Fig Precision-recall curves of each object class for the best-performing model. (PDF)

S5 Fig Size of krill detection boxes versus annotated boxes for the three test sets. (PDF)

S6 Fig Cropped images from each test set to illustrate immediate causes for false positive (FP) and false negative (FN) detections. (PDF)

S7 Fig Proportion of false positive (FP) detections at different confidence scores for the three test sets. (PDF)

S4 Table Raw data of the error analysis of krill, pelagic shrimp, and gelatinous zooplankton on all three test sets. Columns: image (name of image file), error_type (FP or FN); class (object class according to human annotation or detection); x1, y1, x2, y2 (coordinates of bounding box in pixels); score (confidence score of FP detection); test_set (white, red gain 1.5, red gain 5 test set); directory (folder directory of the image with information of non-maximum suppression IoU and confidence threshold that was applied); score_threshold (optimal score threshold as determined by F1-confidence curve); below_above_score_theshold (label indicating if a FP detection was below or above the optimal score threshold); id (unique ID for each FP or FN bounding box); width (width of the image in pixels); height (height of the image in pixels); sample (“yes” if included in random sample, “no” if not included in random sample for the analysis conducted to produce Fig 11 in the manuscript); contrast (“good”, “average”, “bad” contrast of an object with the background), overlap (“yes”, “no”, overlap with another object), visible_morphological_features (visible morphological features of an object); linked_id (id of a detection or annotation of the same object); cause (immediate cause resulting in a FP or FN label); cause_duplicate (cause leading to a duplicate detection); cause_misclassified (cause leading to a misclassification); class_2 (correct class of a misidentified object).
(CSV)
