# Supplementary material for: A deep learning-based model for automatic identification of mesopelagic organisms from in-trawl cameras
Source: PLoS One. 2026 Jan 21;21(1):e0340640. doi: 10.1371/journal.pone.0340640 (PMC12822937; doi:10.1371/journal.pone.0340640)
Supplement: S1 Table — (PDF) [file pone.0340640.s001.pdf]

S1 Table. Number of manual annotations for pelagic shrimp, gelatinous zooplankton and fish coloured according to the original labels. *The number of manual annotations is shown for each of the manually annotated datasets separately: W, R1.5, R5.*

		Manually annotated dataset		
Object class	Original labels	**W**	**R1.5**	**R5**
Pelagic shrimp	*Pasiphaea* spp.	25	0	224
	Eusergestes arcticus	331	0	303
Gelatinous zooplankton	Gelatinous zooplankton	0	44	563
	Periphylla periphylla	1257	0	0
Fish	*Micromesistius poutassou*	842	544	0
	Mallotus villosus	333	0	0
	Gadus morhua	2	0	0
	Gadoid	4	0	14
	Sebastes spp.	1056	0	0
	Pollachius virens	29	0	0
