# Supplementary material for: A deep learning-based model for automatic identification of mesopelagic organisms from in-trawl cameras
Source: PLoS One. 2026 Jan 21;21(1):e0340640. doi: 10.1371/journal.pone.0340640 (PMC12822937; doi:10.1371/journal.pone.0340640)
Supplement: S1 Fig — (PDF) [file pone.0340640.s002.pdf]

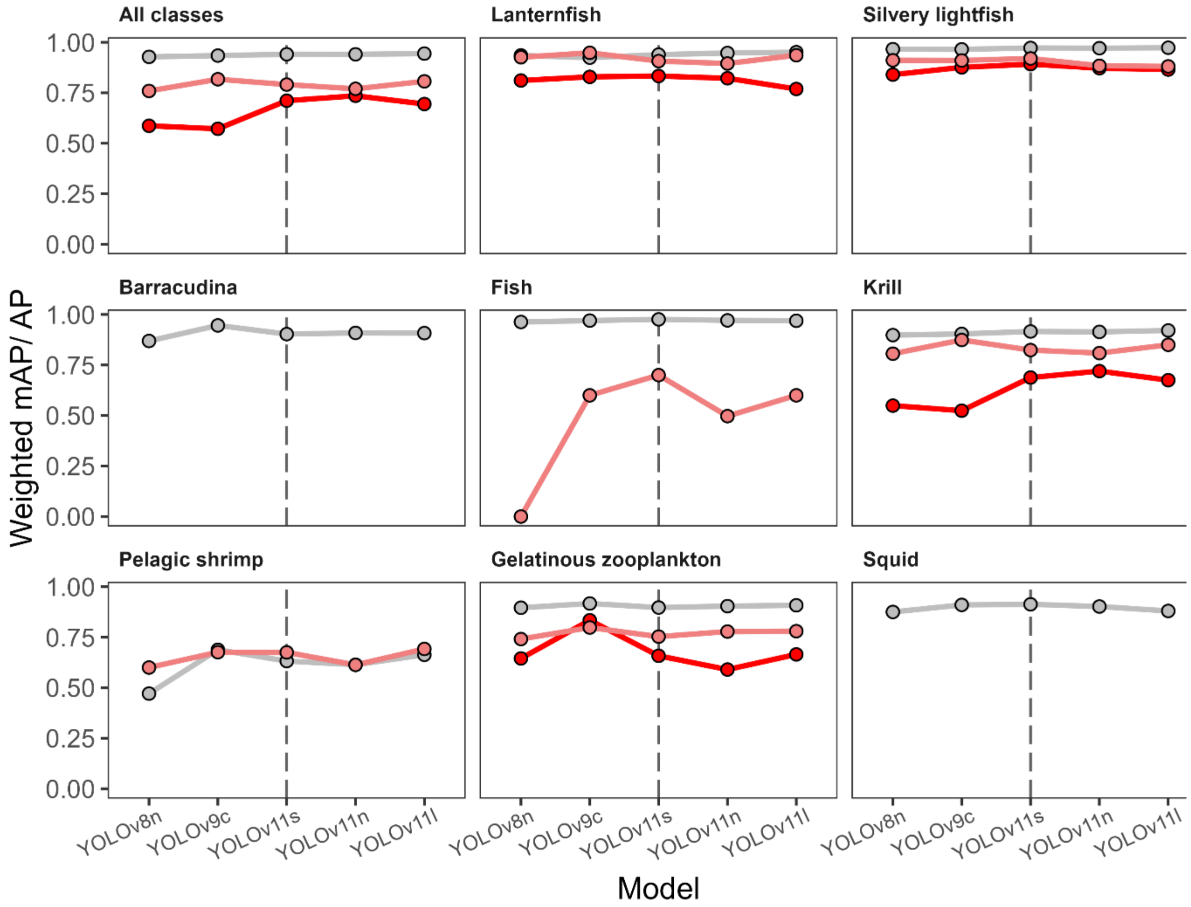

S1 Fig. Experiments to test the effect of YOLO model architecture (v8n, v9c, 11n, 11s, 11l) on the performance of the best-performing model (training set: WRns$_{tr}$, image width: 1216 pixels). *The following parameters used for training, validation and testing, were constant: non-maximum suppression IoU (0.5) and confidence threshold (0.25). The performance of each model is evaluated by testing on white (W$_{te}$, grey), red gain 1.5 (R1.5$_{te}$, red), and red gain 5 (R5$_{te}$, light red) separately and calculating the weighted mean average precision (mAP) and average precision for each object class. The model architecture chosen for this study is YOLO11s (dashed grey line).*
