# Supplementary material for: A deep learning-based model for automatic identification of mesopelagic organisms from in-trawl cameras
Source: PLoS One. 2026 Jan 21;21(1):e0340640. doi: 10.1371/journal.pone.0340640 (PMC12822937; doi:10.1371/journal.pone.0340640)
Supplement: S2 Table — (PDF) [file pone.0340640.s003.pdf]

Five YOLO versions were compared to test the effect of model architecture on the performance of the mesopelagic detector: YOLOv8n, v9c, 11s, 11n, 11l. The models YOLOv9c, YOLO11s and YOLO11l demonstrated the highest performances, with comparable mean average precision scores. Among these, YOLO11s was selected for further use, due to its shorter training time – approximately three times faster than the other two top-performing models. making it a more efficient choice for this study, where we experiment with multiple models.

S2 Table. Comparisons of YOLO models, runtime, accuracy. *The models in this table were trained using an NVIDIA RTX 3090 GPU, on white and red images (WRnstr), with a patience of 20 epochs. Other parameters: image size: 1216, agnostic_nms=True, Mininum confidence threshold=0.25. The model architecture chosen for this study is YOLO11s (bold).*

		Validation	Test				
Model	Batch size	WR_{va}	W_{te}	$R1.5_{te}$	$R5_{te}$	Runtime (h)	Num epochs
YOLO8n	16	0.877	0.859	0.664	0.711	6.17	157
YOLOv9c	4	0.883	0.903	0.801	0.766	16.92	97
YOLO11n	16	0.892	0.892	0.746	0.751	7.33	145
**YOLO11s**	**8**	**0.899**	**0.893**	**0.796**	**0.768**	**5.65**	**92**
YOLO11l	4	0.89	0.897	0.79	0.743	13.75	134
