# Supplementary material for: A deep learning-based model for automatic identification of mesopelagic organisms from in-trawl cameras
Source: PLoS One. 2026 Jan 21;21(1):e0340640. doi: 10.1371/journal.pone.0340640 (PMC12822937; doi:10.1371/journal.pone.0340640)
Supplement: S3 Table — (PDF) [file pone.0340640.s004.pdf]

S3 Table: Model parameters used during training as specified by Ultralytics [1]. *Default values were used for most parameters, except where indicated in bold characters in the value column. Augmentation parameters are specified in the bottom part of the table (separated by a double line).*

Argument	Value	Argument	Value	Argument	Value
model	**yolo11s.pt**	amp	True	mask_ratio	4
epochs	**200**	fraction	1.0	dropout	0.0
patience	**10**	profile	False	val	True
batch	**4**	freeze	None	hsv_h	0.015
imgsz	**1216**	lr0	0.01	hsv_s	0.7
cache	10	lrf	0.01	hsv_v	0.4
device	None	momentum	0.937	degrees	0.0
workers	8	weight_decay	0.0005	translate	0.1
pretrained	True	warmup_epochs	3.0	scale	0.5
optimizer	'auto'	warmup_momentum	0.8	shear	0.0
seed	0	warmup_bias_lr	0.1	perspective	0.0
deterministic	True	box	7.5	flipud	0.0
single_cls	False	cls	0.5	fliplr	0.5
classes	None	dfl	1.5	bgr	0.0
rect	False	pose	12.0	mosaic	1.0
multi_scale	False	kobj	1.0	mixup	0.0
cos_lr	False	nbs	64	cutmix	0.0
close_mosaic	10	overlap_mask	True		
