# Supplementary material for: A deep learning-based model for automatic identification of mesopelagic organisms from in-trawl cameras
Source: PLoS One. 2026 Jan 21;21(1):e0340640. doi: 10.1371/journal.pone.0340640 (PMC12822937; doi:10.1371/journal.pone.0340640)
Supplement: S2 Fig — (PDF) [file pone.0340640.s005.pdf]

Detections with confidence scores below the specified threshold are not taken into account in the calculation of average precision (AP) and mean average precision (mAP). The default value of confidence threshold is set at 0.25 [1]. To evaluate the impact of threshold on model performance, we compared results using thresholds of 0.25 and 0.05. For the two red light datasets, lowering the confidence threshold to 0.05 led to improved mAP and AP values for krill and gelatinous zooplankton.

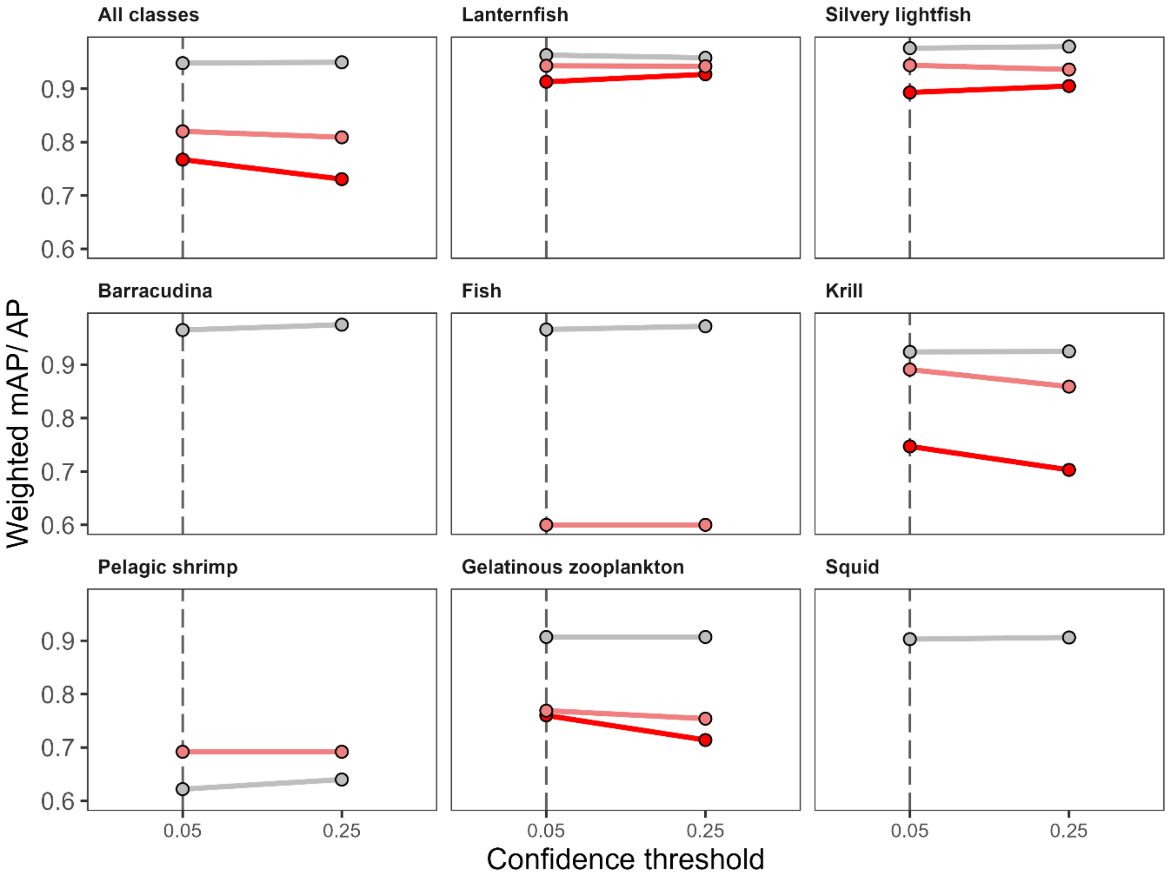


***S2 Fig. Experiments to test the effect of confidence threshold (0.25, 0.05) on the performance of the best-performing model (training set: WRns$_{tr}$, image width: 1216 pixels).*** *The following parameters used for training, validation and testing, were constant: model architecture (YOLO11s), non-maximum suppression IoU (0.4). The performance of each model is evaluated by testing on white (W$_{te}$, grey), red gain 1.5 (R1.5$_{te}$, red), and red gain 5 (R5$_{te}$, light red) separately and calculating the weighted mean average precision (mAP) and average precision for each object class. The confidence threshold chosen for this study is 0.05 (dashed grey line).*


[1] Ultralytics. Configuration n.d. https://docs.ultralytics.com/usage/cfg (accessed September 29, 2025).
