# Supplementary material for: A deep learning-based model for automatic identification of mesopelagic organisms from in-trawl cameras
Source: PLoS One. 2026 Jan 21;21(1):e0340640. doi: 10.1371/journal.pone.0340640 (PMC12822937; doi:10.1371/journal.pone.0340640)
Supplement: S3 Fig — (PDF) [file pone.0340640.s006.pdf]

NMS is a post-processing step to remove overlapping bounding boxes that predict the same object [1]. This is achieved in three steps: First, all bounding boxes with a lower confidence score than the threshold are removed. Second, the remaining bounding boxes are sorted based on their confidence scores. Third, the bounding box with the highest confidence score is compared with all other bounding boxes, by calculating their intersection over union (IoU). All bounding boxes that have a higher IoU than the predefined threshold are suppressed. The last two steps are repeated until no more bounding boxes are removed.

During testing, we explored the effect of varying the threshold IoU for NMS between 0.4 and 0.7. Lowering the IoU threshold minimizes duplicate detections, as detections must be further apart to be considered as separate objects. A threshold IoU of 0.4 was chosen since the generic fish, and gelatinous zooplankton performed slightly better with a lower IoU.

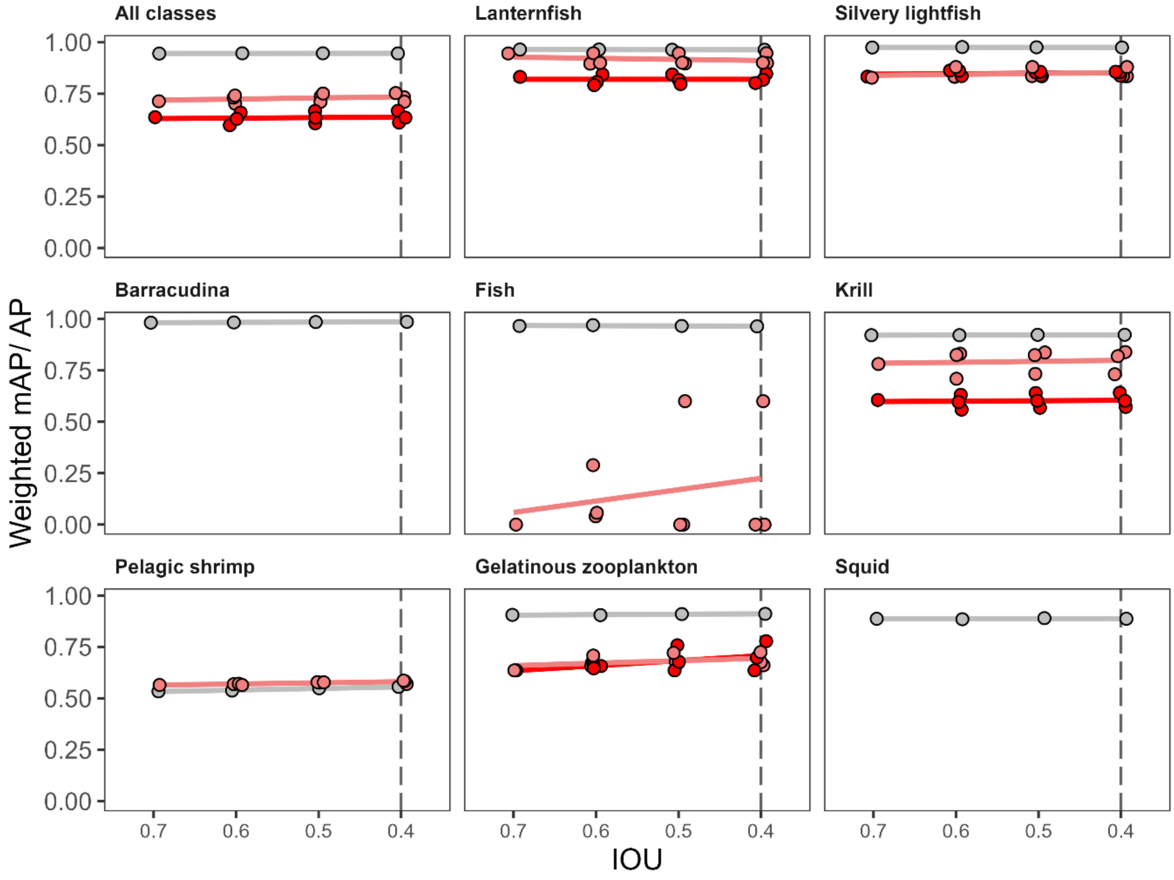

S3 Fig. Experiments to test the effect of threshold intersection over union (IoU) (0.7, 0.6, 0.5, 0.4) during non-maximum suppression on the performance of the best-performing model (training set: WRns$_{tr}$, image width: 1216 pixels). *The following parameters used for training, validation and testing, were constant: model architecture (YOLO11s), confidence threshold (0.05). The performance of each model is evaluated by testing on white (W$_{te}$, grey), red gain 1.5 (R1.5$_{te}$, red), and red gain 5 (R5$_{te}$, light red) separately and calculating the weighted mean average precision (mAP) and average precision for each object class. The threshold IoU chosen for this study is 0.4 (dashed grey line).*

[1] Vijayakumar A, Vairavasundaram S. YOLO-based Object Detection Models: A Review and its Applications. Multimed Tools Appl 2024;83:83535–74. https://doi.org/10.1007/s11042-024-18872-y.
