# Supplementary material for: A deep learning-based model for automatic identification of mesopelagic organisms from in-trawl cameras
Source: PLoS One. 2026 Jan 21;21(1):e0340640. doi: 10.1371/journal.pone.0340640 (PMC12822937; doi:10.1371/journal.pone.0340640)
Supplement: S4 Fig — (PDF) [file pone.0340640.s007.pdf]

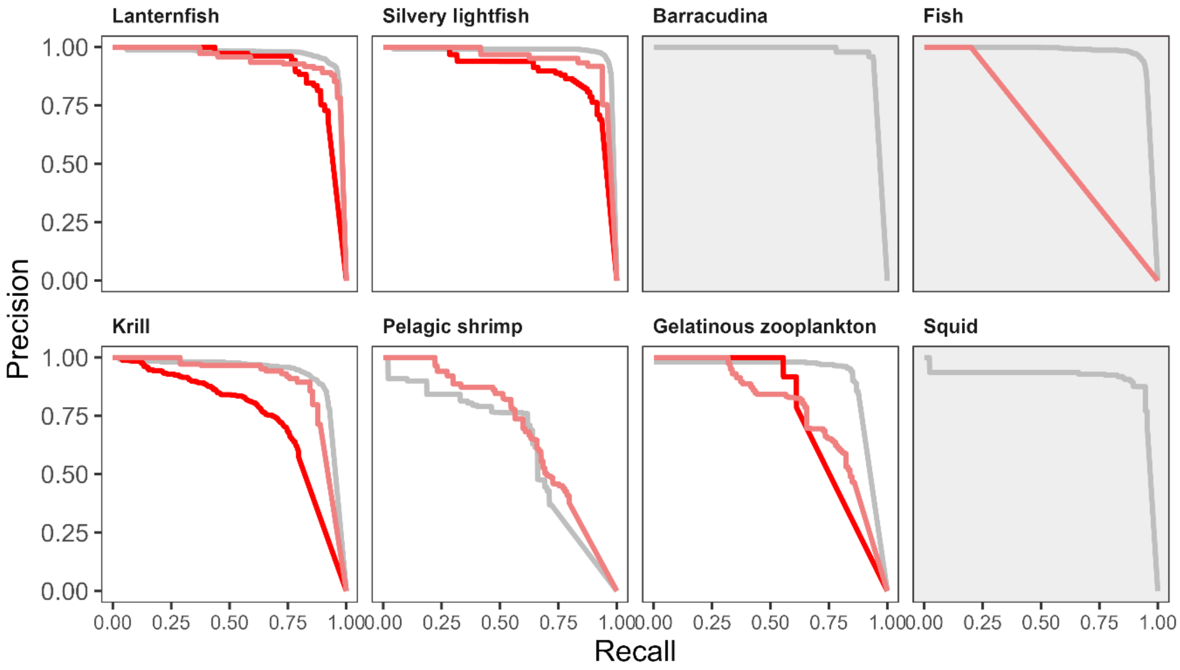

S4 Fig. Precision-recall curves of each object class for the best-performing model (training set: $WRns_{tr}$, image width: 1216 px). *The model was tested separately on white (W_{te}, grey), red gain 1.5 ($R1.5_{te}$, red), and red gain 5 ($R5_{te}$, light red) images. The object classes with a grey background were excluded from the analysis since they were present in only one of the test sets or had too few annotations.*
