# Supplementary material for: A deep learning-based model for automatic identification of mesopelagic organisms from in-trawl cameras
Source: PLoS One. 2026 Jan 21;21(1):e0340640. doi: 10.1371/journal.pone.0340640 (PMC12822937; doi:10.1371/journal.pone.0340640)
Supplement: S5 Fig — (PDF) [file pone.0340640.s008.pdf]

As the krill size wasn't directly measured, we calculated the diagonal of both the annotated and predicted bounding boxes using the Pythagorean theorem. While the test set of white and red gain 1.5 images mainly contained small bounding boxes (<100 px), bounding boxes on red gain 5 images were generally larger (100 to 300 px).

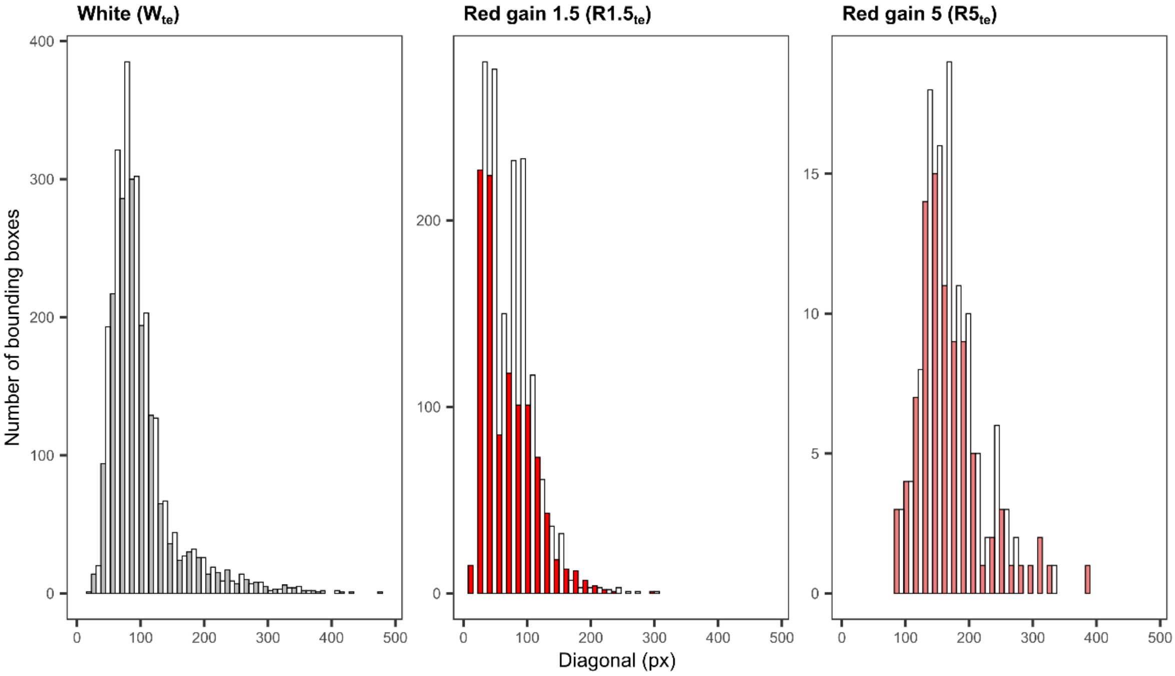

S5 Fig. Size of krill detection boxes versus annotated boxes for the three test sets: white (W_{te}), red gain 1.5 ($R1.5_{te}$), red gain 5 ($R5_{te}$). *Number of detection boxes (white) were produced with the best-performing model (training set: $WRns_{tr}$, image width: 1216 px). Annotation boxes are coloured with respect to the test dataset (grey for images collected under white light, red for red gain 1.5, and light red for red gain 5 images). The size of the krill was estimated using the length of the diagonal of the bounding boxes in pixels (px).*
