# Supplementary material for: A deep learning-based model for automatic identification of mesopelagic organisms from in-trawl cameras
Source: PLoS One. 2026 Jan 21;21(1):e0340640. doi: 10.1371/journal.pone.0340640 (PMC12822937; doi:10.1371/journal.pone.0340640)
Supplement: S6 Fig — (PDF) [file pone.0340640.s009.pdf]

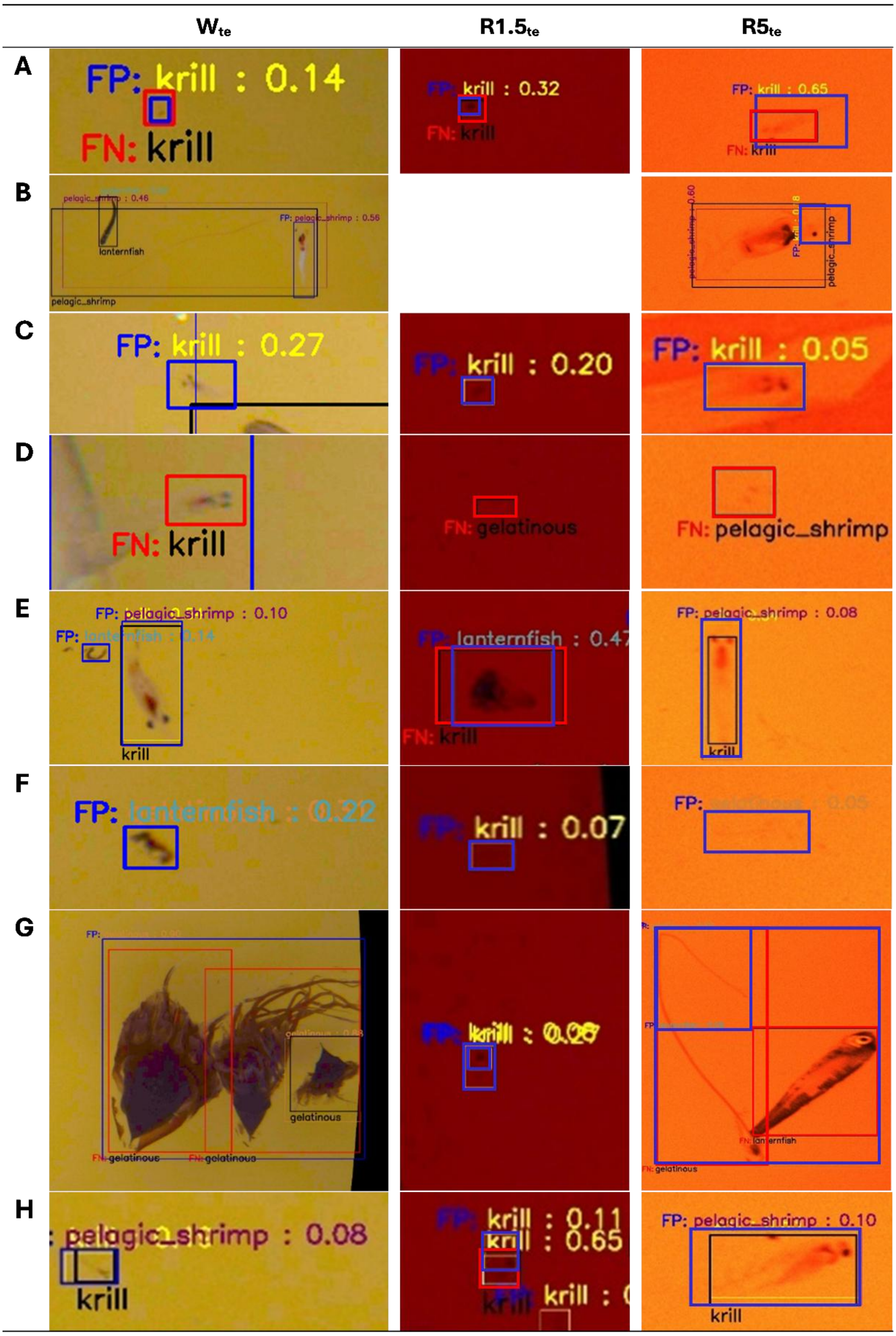

S6 Fig. Cropped images from each test set to illustrate causes for false positive (FP) and false negative (FN) detections: *white (W_{te}, left column), red gain 1.5 ($R1.5_{te}$, middle column), and red gain 5 ($R5_{te}$, right column). Images are overlain with FP automatic detections (red) generated by the best-performing model (training set: WRnstr, image width: 1216 pixels). Each row illustrates one of the immediate causes for FPs and FNs defined in Table 5: (A) mismatch, (B) partially detected object, (C) missed annotation, (D) missed detection, (E) misclassified, (F) unidentifiable object, (G) detection around multiple objects, (H) duplicate detection.*
