# Supplementary material for: A deep learning-based model for automatic identification of mesopelagic organisms from in-trawl cameras
Source: PLoS One. 2026 Jan 21;21(1):e0340640. doi: 10.1371/journal.pone.0340640 (PMC12822937; doi:10.1371/journal.pone.0340640)
Supplement: S7 Fig — (PDF) [file pone.0340640.s010.pdf]

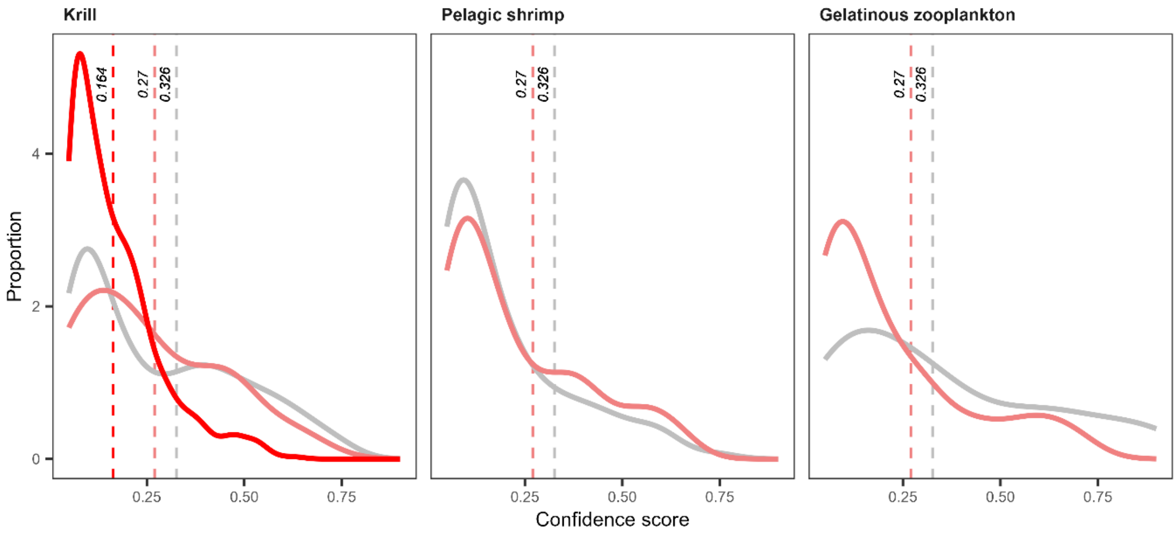

S7 Fig. Proportion of false positive (FP) detections at different confidence scores for the three test sets: *white (W_{te}, grey), red gain 1.5 ($R1.5_{te}$, red), red gain 5 ($R5_{te}$, light red). Dashed vertical lines with labels indicate the optimal confidence threshold for each test set, based on the F1-confidence curves (see Fig 9 in manuscript).*
